# Synergetic Action of Domain II and IV Underlies Persistent Current Generation in Na_v_1.3 as revealed by a tarantula toxin

**DOI:** 10.1038/srep09241

**Published:** 2015-03-18

**Authors:** Cheng Tang, Xi Zhou, Yunxiao Zhang, Zhaohua xiao, Zhaotun Hu, Changxin Zhang, Ying Huang, Bo Chen, Zhonghua Liu, Songping Liang

**Affiliations:** 1 College of Life Science, Hunan Normal University, Changsha, Hunan. 410081, China; 2 Department of Pediatrics, Xiangya Hospital of Central South University, Changsha, Hunan. 410008, China

## Abstract

The persistent current (I_NaP_) through voltage-gated sodium channels enhances neuronal excitability by causing prolonged depolarization of membranes. Na_v_1.3 intrinsically generates a small I_NaP_, although the mechanism underlying its generation remains unclear. In this study, the involvement of the four domains of Na_v_1.3 in I_NaP_ generation was investigated using the tarantula toxin α-hexatoxin-MrVII (RTX-VII). RTX-VII activated Na_v_1.3 and induced a large I_NaP_. A pre-activated state binding model was proposed to explain the kinetics of toxin-channel interaction. Of the four domains of Na_v_1.3, both domain II and IV might play important roles in the toxin-induced I_NaP_. Domain IV constructed the binding site for RTX-VII, while domain II might not participate in interacting with RTX-VII but could determine the efficacy of RTX-VII. Our results based on the use of RTX-VII as a probe suggest that domain II and IV cooperatively contribute to the generation of I_NaP_ in Na_v_1.3.

Voltage-gated sodium channels (Na_v_s) are essential for the initiation and propagation of action potentials in excitable tissues such as nerves and muscles^1^,^2^,^3^. They consist of a pore forming α-subunit (260 kDa) associated with auxiliary β-subunits (30–40 kDa)^4^. In mammals, nine α-subtypes (Na_v_1.1–1.9) have been identified and cloned^5^,^6^. The α-subunit is composed of four homologous domains (DI–DIV) and each domain contains six transmembrane segments (S1–S6). The S5–S6 segments form a central pore for Na^+^, with the S1–S4 segments from each domain forming the surrounding voltage sensors^2^,^4^. Each of the four voltage sensors is activated in response to depolarization of membrane potential^7^; the voltage sensors of the first three domains (DI–III) are responsible for channel activation, while that of the fourth domain (DIV) determined fast inactivation^8^,^9^. Na_v_s fail to inactivate in some cases, resulting in the generation of non-inactivating persistent Na^+^ currents (I_NaP_), which account for up to 5% of the transient peak inward current (I_NaT_) in physiological conditions^10^,^11^. Despite its small amplitude compared with I_NaT_, I_NaP_ amplifies synaptic potentials and aids in the repetitive firing of action potentials (AP) in neurons because it is activated by sub-threshold voltages^11^. The voltage range in which I_NaP_ is activated is traversed by interspike intervals during an AP train^11^,^12^. I_NaP_ has been characterized in a variety of neurons^13^. Mutations in some Na_v_s genes causing enhanced I_NaP_ amplitude are correlated to diseases, such as heart vascular disease and epilepsy^11^,^14^,^15^. Therefore, drugs targeting I_NaP_ are expected to have therapeutic benefits^16^,^17^,^18^.

The early understanding of the origin of I_NaP_ is based on three hypotheses: (1) the window current hypothesis^19^; (2) I_NaP_ is generated by unusual subtypes of Na_v_s which lacking inactivation^20^,^21^; (3) I_NaP_ is produced by the same population of channels responsible for I_NaT_ through a distinct gating mechanism^22^,^23^. Many lines of evidences support the third hypothesis, namely that some channels of a specific Na_v_ subtype occasionally enter the late brief opening and burst of opening state in single channel recordings^24^,^25^,^26^. The single channel mechanism for the slowed inactivation of a Na_v_ by site 3 toxins TsIV-5 and anthopleurin B was also attributed to the increment of mean open time as well as prolonged bursting^27^,^28^; studies showed that mutations in all four domains of Na_v_s as well as intracellular loops affect the amplitude of the I_NaP_^29^; wild type (wt)-Na_v_1.3 naturally develops I_NaP_ in neonatal and axotomized neurons^30^,^31^, and Na_v_1.3 expressed in HEK293T cells displays a clearly detectable I_NaP_^32^,^33^. These data also confirm that I_NaP_ is the intrinsic property of Na_v_s themselves, although the molecular determinants of I_NaP_ in Na_v_s are largely unknown.

In the present study, we identified a tarantula toxin named α-hexatoxin-MrVII (RTX-VII) that enhances the I_NaP_ of Na_v_1.3 and used it as a probe to examine the involvement of each domain of Na_v_1.3 in the generation of I_NaP_. Our results reveal that domains II and IV work in a synergetic manner to determine the toxin-induced I_NaP_ of Na_v_1.3.

## Results

### RTX-VII enhances the I_NaP_ of Na_v_1.3

**Figure 1:**
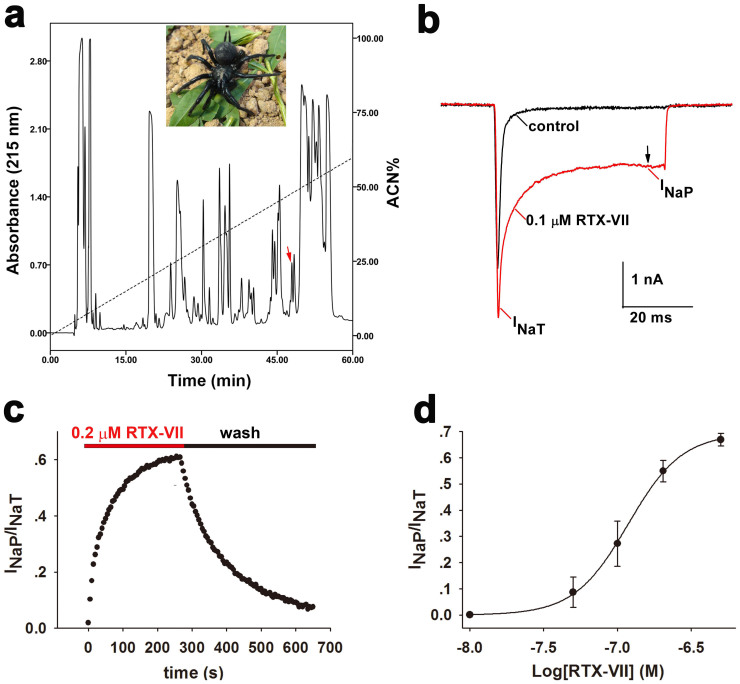
RTX-VII induces large I_NaP_ in Na_v_1.3. (a) RP-HPLC profile of the venom from the spider *Macrothele raveni* (*inset, photo by Dr Songping Liang*), where red arrow indicates the fraction containing RTX-VII. (b) Current traces from a representative cell show 0.1 μM RTX-VII enhances the I_NaT_ of Na_v_1.3 and induces a large I_NaP_ at the end of a 50-ms depolarization to −10 mV from a holding potential of −100 mV (n = 6). Note I_NaP_ was measured at the time point of 45 ms. (c) Time course for the enhancement of I_NaP_ of Na_v_1.3 by 0.2 μM RTX-VII and the recovery upon washing with bath solution, τ_on_ and τ_off_ is 40.9 ± 11.3 s and 162.8 ± 39.7 s, respectively (n = 4). (d) RTX-VII dose dependently enhances I_NaP_ of Na_v_1.3 with an apparent EC_50_ of 0.12 μM (n = 6). The maximum response (f_max_) and the minimum response (f_min_) of Na_v_1.3 to RTX-VII is 66.90% and 0, respectively.

*Macrothele raveni* (Figure 1a, *inset*) venom was collected by using an electro-pulse stimulator as described previously^34^. The lyophilized crude venom was fractionated by RP-HPLC (Figure 1a). A comprehensive screening of each eluted fraction against Na_v_1.3 transiently expressed in HEK293T cells indicated that the fraction with a retention time of 44.6 min inhibited the fast inactivation of this channel (Figure 1b). This fraction contained a peptide with a molecular weight of 4064.71 Da as determined by MALDI-TOF MS, which was then further purified by analytical RP-HPLC (Supplementary Fig. S1a). Sequence of the peptide was determined by combining Edman degradation (Supplementary Fig. S1j) and cDNA sequencing (Supplementary Fig. S1b), and the toxin was named α-hexatoxin-MrVII (RTX-VII). Blasting the full amino acid sequence of RTX-VII showed that it share 92% identity to a previously known spider toxin Magi-6 (Supplementary Fig. S1c). However, Magi-6 did not compete with the scorpion toxin LqhαIT in binding site 3 of Na_v_s, and the symptoms caused by injection of pure Magi-6 to mice could not be directly linked to a particular ion channel receptor^35^. This raise the possibility that the subtle amino acid sequence variation brought by RTX-VII makes it active on mammalian Na_v_s, as that of APETx3 and APETx1, in which a single amino acid substitution between them confer these two toxins different ion channel selectivity^36^. RTX-VII contains eight cysteine residues forming four disulfide bonds, as the measured molecular weight was 8 Da less than the theoretical one. The conserved arrangement of the cysteine residues in RTX-VII indicated that it contains a cystine knot (ICK) motif (Supplementary Fig. S1c).

As shown in Figure 1b, RTX-VII had three effects on current of Na_v_1.3: (1) it increased the I_NaT_ amplitude at the depolarizing voltage of −10 mV; (2) it inhibited the fast inactivation of the channel as determined by *I_5ms_/I_NaT_* ratio; (3) and it induced a large I_NaP_ as revealed by the *I_45ms_/I_NaT_* ratio. At a depolarization of −10 mV, the I_NaP_ generated by Na_v_1.3 accounted for little of the I_NaT_ under control conditions, whereas the treatment with 0.1 μM RTX-VII enhanced the I_NaP_ to approximate 25% of the I_NaT_. The I_NaP_ evoked by the toxin lasted for several seconds and large tail current was observed when cell membrane was repolarized (Supplementary Fig. S1d), distinguishing this toxin from certain α-scorpion toxins. The time course for 0.2 μM RTX-VII activating the I_NaP_ of Na_v_1.3 was characterized by a slow onset of action (τ_on_ = 40.9 ± 11.3 s) and a slow recovery upon washing (τ_off_ = 162.8 ± 39.7 s) (Figure 1c). The activation of the I_NaP_ of Na_v_1.3 by RTV-VII was dose-dependent, with an apparent EC_50_ of 120 nM (Figure 1d). The activity and selectivity of RTX-VII were examined against three Na_v_ subtypes (Na_v_1.4, 1.5 and 1.7) expressed in HEK293T cells, TTX-R Na_v_s of rat dorsal root ganglion neurons and Na_v_s in neonatal rat hippocampal neurons. Among these channels, Na_v_s of neonatal rat hippocampal neurons were sensitive to this toxin (Supplementary Fig. S1i), whereas the others were not (Supplementary Fig. S1e–h).

### Kinetics of RTX-VII action on Na_v_1.3

**Figure 2:**
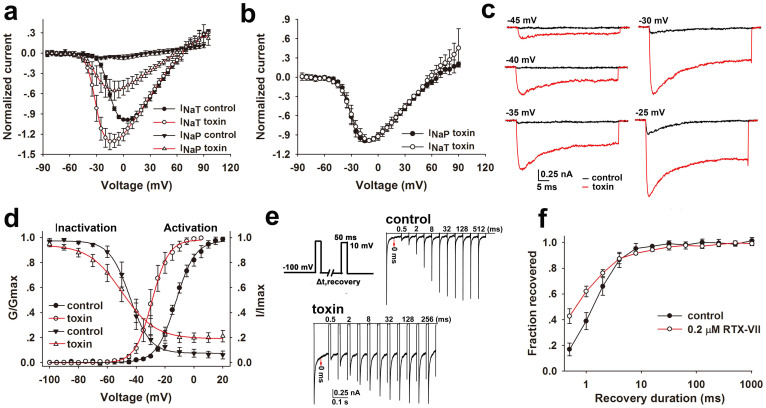
Kinetics for RTX-VII interacting with Na_v_1.3. (a) I–V curves of the I_NaP_ and I_NaT_ of Na_v_1.3 before and after 0.2 μM RTX-VII treatment. Each current component was normalized to the maximum I_NaT_ in control (n = 11). (b) I-V curves of the I_NaT_ and I_NaP_ of Na_v_1.3 in the presence of 0.2 μM RTX-VII. The I_NaP_ and I_NaT_ at each depolarizing voltage were normalized to their maximum one, respectively (n = 11). (c) A cluster of current traces from a representative cell show that 0.2 μM RTX-VII enhances the I_NaT_ and I_NaP_ of Na_v_1.3 at various depolarizing voltages (n = 11). (d) 0.2 μM RTX-VII causes a hyperpolarized shift of the steady-state activation curve of Na_v_1.3 without changing the slope factor (*V_a_* = −12.72 ± 4.92 mV for control and *V_a_* = −29.64 ± 6.21 mV for the toxin treatment; *K_a_* = 6.83 ± 0.98 mV for control and *K_a_* = 5.66 ± 1.12 mV for the toxin treatment) (n = 11), as well as a small but significant hyperpolarized shift of the steady-state inactivation accompanied by a significant change of the slope factor (*V_h_* = −44.58 ± 2.73 mV for control and *V_h_* = −51.12 ± 5.00 for the toxin treatment, p < 0.05; *K_a_* = −6.98 ± 1.44 mV for control and *K_a_* = −12.49 ± 0.41 mV for the toxin treatment, p < 0.001) (n = 5). (e) Representative traces shows the recovery of Na_v_1.3 from fast inactivation before (*control*) and after (*toxin*) 0.2 μM RTX-VII treatment. The red arrow indicates the recovery duration of 0 ms; the numbers labeled above the traces show the recovery time. The repriming protocol is also shown (n = 5). (f) Time-dependent recovery of Na_v_1.3 from fast inactivation in the presence and absence of 0.2 μM RTX-VII (n = 5).

The current-voltage (I–V) relationships of the I_NaT_ and I_NaP_ of Na_v_1.3 before and after the application of 0.2 μM RTX-VII were explored (Figure 2, Supplementary Fig. S2). Compared with the control, RTX-VII modified the I–V relationship of the I_NaT_ as follows: (1) the activation of the I_NaT_ was potentiated by the toxin at voltages ranging from −45 mV to 10 mV, while no potentiation was observed at voltages > 10 mV (Figure 2a); (2) the activation voltage of the maximum I_NaT_ was shifted from 5 mV in the control to −10 mV in the presence of the toxin (Figure 2a, Supplementary Fig. S2d). Although RTX-VII did not alter the reversal voltages (approximate 65 mV) of Na_v_1.3, toxin application did negatively shift channels' initial activation voltage (Figure 2a and c). These data indicate that the toxin treatment may increase the opening probability of Na_v_1.3 channels in cell membrane and facilitate their activation at weak depolarizing voltages. The I–V curves of the I_NaP_ before and after the application of toxin indicate that the enhancement of the I_NaP_ by the toxin occurred across the depolarizing voltages tested (Figure 2a, Supplementary Fig. S2a) and the activation voltage of the maximum I_NaP_ was at about −10 mV. Regarding the I–V curves of the I_NaP_ and I_NaT_ in the presence of toxin, if the amplitudes of the I_NaP_ and I_NaT_ at each depolarizing voltage were normalized to their maximum one, respectively, they overlapped completely (Figure 2b), suggesting that the I_NaP_ and I_NaT_ in the presence of the toxin share rather similar activation voltage. Similar to some α-scorpion toxins^37^, RTX-VII removed the fast inactivation of Na_v_1.3 in a voltage-independent way at depolarizing voltages ranging from −20 mV to +30 mV (Supplementary Fig S2b).

The conductance-voltage (G–V) relationship and the steady-state inactivation of Na_v_1.3 before and after the application of RTX-VII were explored (Figure 2d). Compared with the control, RTX-VII increased the conductance of the cell membrane at depolarizing voltages below 10 mV as revealed by an approximate 16 mV negative shift of Na_v_1.3 channels' activation curve induced by the toxin (*V_a_* = −12.72 ± 4.92 mV for control and *V_a_* = −29.64 ± 6.21 mV for the toxin treatment), which is in accordance with the negative shift of the activation voltage for maximum I_NaT_ observed in the I–V curve; the toxin did not significantly alter the slope factor of the activation curve (*K_a_* = 6.83 ± 0.98 mV for control and *K_a_* = 5.66 ± 1.12 mV for the toxin treatment). The I–V and G–V relationships of Na_v_1.3 before and after RTX-VII application were acquired with stringent controls of the uncompensated series resistance (Rs) caused depolarizing voltage error (the maximum tolerable voltage error was less than 5 mV, the mean maximum Rs-caused voltage error was 2.34 ± 1.08 mV, p < 0.001 when compared to the *Va* shifted amplitude). A steady-state component (approximate 20% of the I_NaT_) that was resistant to inactivation was observed in the steady-state inactivation (SSI) curve when conditional voltages were above −20 mV, which should represent the I_NaP_ elicited by conditional pulses. A significant change of *V_h_* and *K_h_* were observed (*V_h_* = −44.58 ± 2.73 mV for control and *V_h_* = −51.12 ± 5.00 mV for the toxin treatment, p < 0.05; *K_h_* = −6.98 ± 1.44 mV for control and *K_h_* = −12.49 ± 0.41 mV for the toxin treatment, p < 0.001). The hyperpolarization shift of the G–V curve and a non-inactivated component in the SSI curve together resulted in an enlarged voltage range for generation of window current, indicating a slower development of closed state inactivation (CSI) in the toxin-treated channels.

The effect of RTX-VII on the repriming kinetics (recovery from fast inactivation) of Na_v_1.3 was also investigated. As shown in Figure 2e, the I_NaT_ of Na_v_1.3 recovered gradually from fast inactivation with the repolarizing time (recovery time) increasing in the absence (*control*) and presence (*toxin*) of RTX-VII. The I_NaP_ induced by the toxin was observed at all recovery time. The I_NaP_ of the toxin-treated channels fully recovered at the recovery time of 0 ms, but no I_NaT_ recovery was observed (Figure 2e, *toxin*). The recovery ratios of Na_v_1.3 I_NaT_ before and after the application of toxin were plotted as a function of recovery time (Figure 2f), showing most of channels (>80%) recovered from fast inactivation in 4 ms in both conditions. An apparently faster repriming of the toxin-treated channels than that of control channels within 4 ms was observed, which could be associated with the existence of the I_NaP_. If the I_NaP_ was subtracted from the I_NaT_ in toxin treated channels, the residual current would exhibit the same repriming kinetics as that of the control (Supplementary Fig. S2c).

### The molecular mechanism of RTX-VII as an excitatory toxin

**Figure 3:**
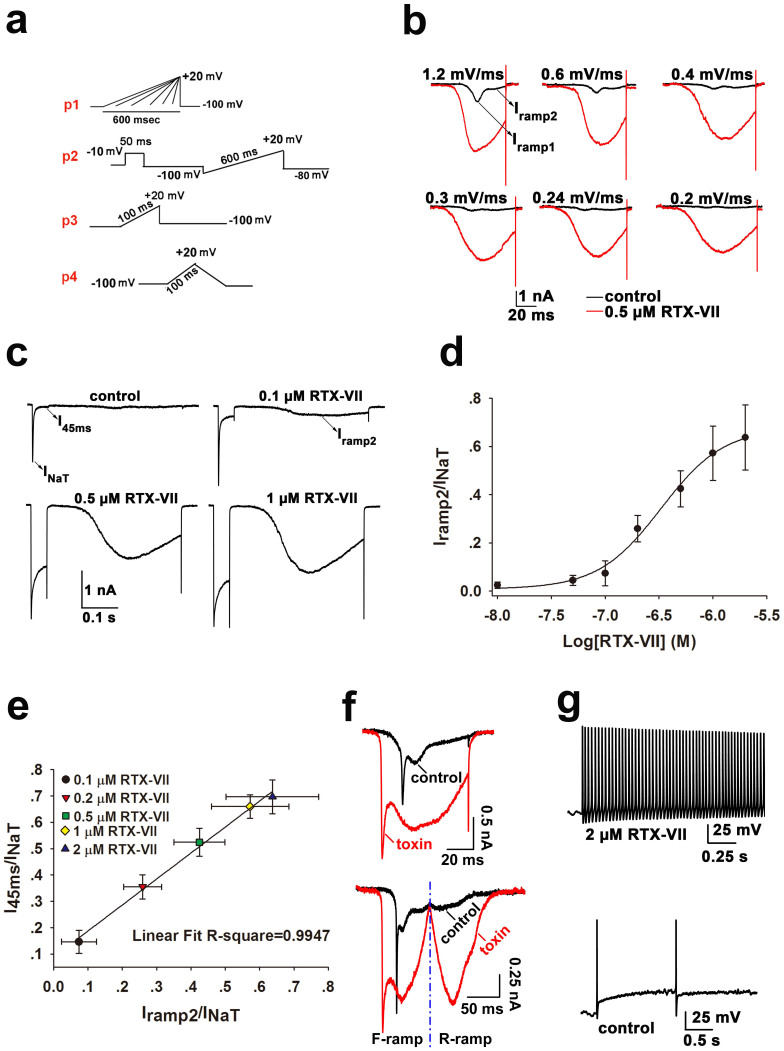
The effects of RTX-VII on the ramp currents of Na_v_1.3 and Na_v_s of hippocampal neurons. (a) Protocols are used in the experiments described in this figure. (b) A series of inward Na_v_1.3 currents are evoked by using linearly increasing ramp voltage from −100 mV to 20 mV with different ramp rate (Figure 3a, p1), the ramp time ranges from 100 ms to 600 ms, 100 ms/step. The Na_v_1.3 ramp current (I_ramp_) displays two peaks with the first one (I_ramp1_) but not the second one (I_ramp2_) being sensitive to ramp rate in control (*black traces*). 0.5 μM RTX-VII enhances the amplitudes of both peaks and causes a hyperpolarized shift of the initial activation voltage for I_ramp1_ (*red traces*); numbers labeled above the traces indicate the ramp rate (mV/ms) (n = 10). (c) Representative traces show that RTX-VII dose-dependently enhances the I_NaP_ (I_45ms_) and I_ramp2_ of Na_v_1.3 elicited by the protocol p2 shown in Figure 3a (n = 5). (d) Dose-response curve for RTX-VII activating the I_ramp2_ of Na_v_1.3, the apparent EC_50_ is determined as approximate 0.3 μM; the maximum and the minimum response of Na_v_1.3 to RTX-II is 63.03% and 2.15%, respectively (n = 5). (e) The I_45ms_/I_NaT_ ratio was plotted as a function of the I_ramp2_/I_NaT_ ratio at each toxin concentration (data from Figure 3c). A linear fit of the dots shows the close correlation between the I_NaP_ and I_ramp2_ of Na_v_1.3 (R^2^ = 0.9947) (n = 5). (f) Compared with control, 1 μM RTX-VII evidently enhances both peaks (I_ramp1_ and I_ramp2_) of the ramp current of Na_v_s in rat hippocampal neurons (*upper*). Protocol p3 shown in Figure 3a was used (n = 5); Representative traces (*below*) show that the I_ramp_ of Na_v_s in hippocampal neurons is elicited by protocol p4 shown in Figure 3a (n = 5). (g) Spontaneous AP firing in a neonatal rat hippocampal neurons in the absence (*below*) and presence (*upper*) of 2 μM RTX-VII (n = 8).

The enhancement of I_NaP_ of Na_v_s in hippocampal neurons by RTX-VII may have led to excitatory toxic in mouse. Intracerebroventricular injection of 20 ng RTX-VII dissolved in 20 μl saline caused seizure-like symptoms, as described by circular running in the first several minutes followed by involuntary body twitching, while animals in control group injected with 20 μl saline behaves normal (n = 5 in each group, Supplementary video). We therefore investigated the mechanism of RTX-VII as excitatory toxin. Na_v_1.3 is upregulated in the peripheral nervous system in response to nerve injury, and contributes to the hyperexcitability of nociceptive neurons under neuropathic conditions^38^,^39^. The fast repriming kinetics and slow development of CSI of Na_v_1.3 make it suitable for generating a large response to slowly developing depolarizing inputs (ramp stimuli)^40^. We first tested the effect of RTX-VII on the ramp current (I_ramp_) of Na_v_1.3 evoked by various ramp stimuli (Figure 3a p1). Consistent with previous studies^33^, Na_v_1.3 expressed in HEK293T cells produced a large inward Na^+^ current in response to a linearly increasing voltage ramp from −100 mV to 20 mV at the ramp rate of 1.2 mV/ms; of the two I_ramp_ peaks shown, the first one (I_ramp1_) but not the second one (I_ramp2_) was ramp rate-dependent, with higher rate leading to larger I_ramp1_ (Figure 3b, *black trace*). The application of 0.5 μM RTX-VII increased the amplitude of both I_ramp1_ and I_ramp2_ generated by Na_v_1.3 at all ramp rates tested along with a hyperpolarization shift of the initial activation voltage for I_ramp1_ (Figure 3b, *red trace*; the maximum tolerable voltage error was less than 5 mV, the mean maximum Rs-caused depolarizing voltage error was 3.45 ± 1.26 mV).The negative shift of I_ramp1_ of Na_v_1.3 was consistent with the channels' negatively shifted activation. The enhanced activation of I_ramp1_ of Na_v_1.3 may have been derived from the larger potential gradient (caused by the negative shift of activation voltage of I_ramp1_) that drives Na^+^ to cross the membrane as well as a slowed CSI which makes more channels available for activation.

Na_v_1.3 intrinsically produces small I_NaP_, and the relationship between I_NaP_ and I_ramp2_ was investigated in a previous study in which a close correlation between them was observed^33^. To clarify the relationship between the RTX-VII evoked I_NaP_ and I_ramp2_, the protocol p2 described in Figure 3a was used to elicit two type currents of Na_v_1.3 (Figure 3c). Note only I_ramp2_ could be evoked at the ramp rate of 0.2 mV/ms (Figure 3b and Figure 3c). RTX-VII dose-dependently enhanced I_NaP_ (I_45ms_) and I_ramp2_ of Na_v_1.3 (Figure 3c).The apparent EC_50_ for RTX-VII activating I_ramp2_ was 320 nM, as revealed by plotting I_ramp2_/I_NaT_ ratios as a function of toxin concentrations (Figure 3d). This EC_50_ value did not differ much from that of RTX-VII activating the I_NaP_ of Na_v_1.3 (120 nM). Furthermore, the correlation coefficient between I_NaP_ and I_ramp2_ was 0.9947 (Figure 3e), indicating a close correlation between them. Thus, data derived from the toxin study further confirmed the conclusion described above.

The effect of RTX-VII on the ramp current of Na_v_s in neonatal hippocampal neurons was also examined. As shown in Figure 3f, both I_ramp1_ and I_ramp2_ of hippocampal Na_v_s were evoked by a linearly increasing voltage ramp from −100 mV to 20 mV at the ramp rate of 1.2 mV/ms (Figure 3a, p3); both components of the I_ramp_ of hippocampal Na_v_s were greatly enhanced by toxin (Figure 3f, *upper*). As shown in Figure 3f (*below*), both I_ramp1_ and I_ramp2_ of hippocampal Na_v_s in control conditions displayed voltage-dependent inactivation by reverse ramp (R-ramp) stimulation following forward ramp (F-ramp) stimulation (Figure 3a, p4); as I_ramp1_ disappeared, the amplitude of I_ramp2_ decreased in the R-ramp compared with that in the F-ramp. On the contrary, 1 μM RTX-VII treatment removed the voltage-dependent inactivation of I_ramp2_ but not I_ramp1_, as toxin induced a nearly unchanged I_ramp2_ in both the forward and reverse ramps, while I_ramp1 _was absent in the R-ramp. This finding indicates that the amplitude of I_ramp2_ in the presence of the toxin is only dependent on the transmembrane potential, and this population of Na_v_s generating I_ramp2_ should maintain a continuous open state during the entire time course of AP. The toxin negatively shifted I_ramp1_ and the enhanced activation of I_ramp2_ might lower the threshold and increase the frequency of AP in hippocampal neurons, respectively, which possibly triggers the spontaneous AP firing in hippocampal neurons at a physiological resting potential. Current-clamp experiments showed that 2 μM RTX-VII triggered spontaneous high frequency AP firing in hippocampal neurons (Figure 3g, *upper*; Supplementary Fig. S3), a mechanism underlying toxin-induced seizure-like symptom in mice. By contrast, the spontaneous AP firing was rare in hippocampal neurons under control conditions (Figure 3g, *below*; Supplementary Fig. S3).

### Domains II and IV of Na_v_1.3 are critical for I_NaP_ generation by RTX-VII

**Figure 4:**
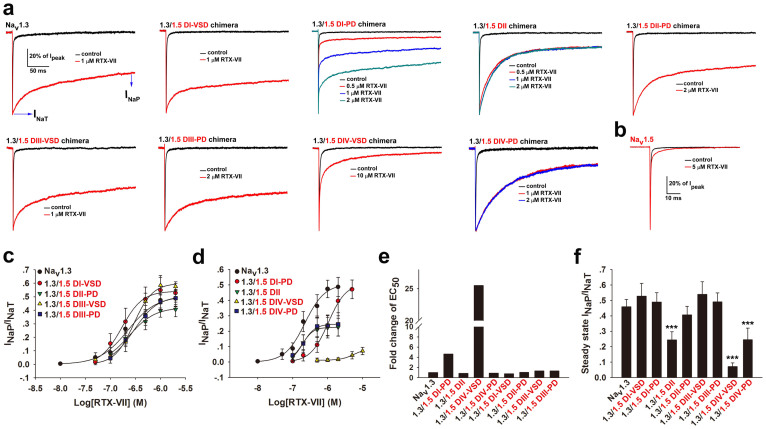
Domain II and IV of Na_v_1.3 are synergistically involved in I_NaP_ generation. (a) A 300-ms recording of currents of wt-Na_v_1.3 and Na_v_1.3 derived chimeric channels before and after the application of various concentration of RTX-VII. Chimeric channels were constructed as follows: the voltage-senor domain (VSD) or pore domain (PD) of DI, DII, DIII or DIV of Na_v_1.3 was substituted with the corresponding domain of Na_v_1.5 (see Supplementary Fig. S4). Here, I_NaP_ was measured at time point of 295 ms. Note DII, DIV-VSD, DIV-PD substitutions in Na_v_1.3 results in the reduction of toxin induced I_NaP_ compared with wt- and other chimeric channels (n = 7–11). (b) Representative traces show that Na_v_1.5 is resistant to RTX-VII (n = 4). (c) Dose-response curves for RTX-VII activating the I_NaP_ of wt-Na_v_1.3 channel and Na_v_1.3 derived chimeric channels that did not or slightly changed toxin potency (EC_50_) or efficacy (steady-state I_NaP_/I_NaT_ ratio at the saturated concentration of the toxin) (n = 7–11). (d) Dose-response curves for RTX-VII activating the I_NaP_ of wt-Na_v_1.3 and chimeric channels that dramatically changed toxin potency and/or efficacy (n = 7–11). (e) Bars show the fold changes of the apparent EC_50_ of RTX-VII on each Na_v_1.3 derived chimeric channel compared with that for wt-Na_v_1.3 (n = 7–11). (f) Bars show ratios of the steady-state I_NaP_/I_NaT_ of wt- and Na_v_1.3 derived chimeric channels in the presence of the saturated concentration of toxin. These values are 45.88 ± 4.78%, 52.78 ± 8.32%, 48.87 ± 6.09%, 24.46 ± 5.27%, 40.64 ± 5.47%, 53.95 ± 8.13%, 49.00 ± 5.88%, 7.16 ± 2.33% and 24.48 ± 7.52% for the chimeric channels Na_v_1.3, 1.3/1.5 DI-VSD, 1.3/1.5 DI-PD, 1.3/1.5 DII, 1.3/1.5 DII-PD, 1.3/1.5 DIII-VSD, 1.3/1.5 DIII-PD, 1.3/1.5 DIV-VSD and 1.3/1.5 DIV-PD, respectively (***p < 0.001, when compared with wt-1.3) (n = 7–11).

Because Na_v_1.5 is resistant to RTX-VII (Figure 4b), a chimera strategy was used to screen the critical modules [voltage sensor domains (VSD) or pore domains (PD)] responsible for the toxin-induced I_NaP_ of Na_v_1.3. Each module from the four domains of Na_v_1.3 was substituted with the corresponding Na_v_1.5 module (Supplementary Fig. S4). The nomenclature of a specific chimeric channel was defined as follows: for example, Na_v_1.3/1.5 DI-VSD chimera is a hybrid channel in which the DI-VSD of Na_v_1.3 was replaced with that of Na_v_1.5. Eight Na_v_1.3 derived chimeric channels were constructed. All chimeric channels except the Na_v_1.3/1.5 DII-VSD chimera were functionally expressed in HEK293T cells; therefore, the hybrid channel Na_v_1.3/1.5 DII was generated instead of the Na_v_1.3/1.5 DII-VSD chimera (Supplementary Fig. S4). To assess the potency and efficacy of RTX-VII for I_NaP_ generation in each wt- or chimeric channel, a 300-ms depolarization to 10 mV from a holding potential of −100 mV was applied to evoke the I_NaT_ and I_NaP_ of a specific channel in the absence and presence of various concentrations of toxin, and the I_NaP_ was measured at the time point of 295 ms (Figure 4a) because the currents of chimeric channels reached a macroscopic steady state at the time point of 300 ms. To compare data derived from different channels, the relative values of I_NaP_/I_NaT_ (both from the same current trace) after treatment with different concentrations of toxin were calculated, and the potency of RTX-VII on a specific channel was defined as the EC_50_ value, while the efficacy of RTX-VII was determined by steady-state I_NaP_/I_NaT_ ratio at the saturated concentration of toxin.

The substitution of the VSD/PD of Na_v_1.3 with that of Na_v_1.5 had different effects on the potency and efficacy of RTX-VII. Compared with the wt-channel, five chimeric channels, namely Na_v_1.3/1.5 DI-VSD, DI-PD, DII-PD, DIII-VSD and DIII-PD produced a large I_NaP_ in response to RTX-VII, whereas the other three chimeric channels, Na_v_1.3/1.5 DII, DIV-VSD and DIV-PD displayed a smaller I_NaP_ (Figure 4a). The apparent EC_50_ values of RTX-VII on these channels were further determined from dose-response curves (Figure 4c and d). The bar diagrams shown in Figure 4e and f indicate the changes in the potency and efficacy of RTX-VII on Na_v_1.3 derived chimeric channels, respectively, which could be described as follows: (1) in the chimeras Na_v_1.3/1.5 DI-VSD, DII-PD, DIII-VSD and DIII-PD, no significant changes in the potency and efficacy of RTX-VII were observed, as indicated by the negligible reduction in the steady-state I_NaP_/I_NaT_ ratios and the less than two-fold increments of EC_50_ values; (2) the chimera Na_v_1.3/1.5 DI-PD generated a large steady-state I_NaP_, similar to that of the wt-Na_v_1.3; however, an approximate 4.8 fold increase of the EC_50_ value was observed, indicating that this chimeric channel reduced the binding affinity of RTX-VII but not the efficacy; (3) the chimeras Na_v_1.3/1.5 DII and DIV-PD significantly decreased the efficacy of RTX-VII, as revealed by a significantly smaller steady-state I_NaP_/I_NaT_ ratios (steady-state I_NaP_ only accounts for approximate 24% of I_NaT_ after 2 μM RTX-VII treatment, P < 0.001 when compared to wt-Na_v_1.3); however, these substitution resulted in a < 2-fold increase of the EC_50_ values; (4) In the chimera Na_v_1.3/1.5 DIV-VSD, both the potency and efficacy of RTX-VII was significantly attenuated, because the toxin, even at a concentration of 10 μM, induced a small fraction of steady state I_NaP_ (<10% of the I_NaT_, P < 0.001, when compared to wt-Na_v_1.3) in this chimeric channel and the apparent EC_50_ of toxin on this chimeric channel was increased by approximate 25 folds when compared to wt-Na_v_1.3. Taken together, these findings suggest that DIV-VSD, DIV-PD, DII and DI-PD of Na_v_1.3 play important roles in the RTX-VII-induced I_NaP_. The different influence of these Na_v_1.3 module substitutions on the potency and efficacy of RTX-VII suggest that they play different roles. DIV-VSD and DI-PD might jointly compose the binding receptor for RTX-VII, whereas the loss of efficacy of RTX-VII on the chimeric channel Na_v_1.3/1.5 DII was not caused by loss of toxin binding but was rather associated with an intrinsic limitation of this hybrid channel in generating a larger I_NaP_.

### Domain II of Na_v_1.3 is not involved in interacting with RTX-VII

**Figure 5:**
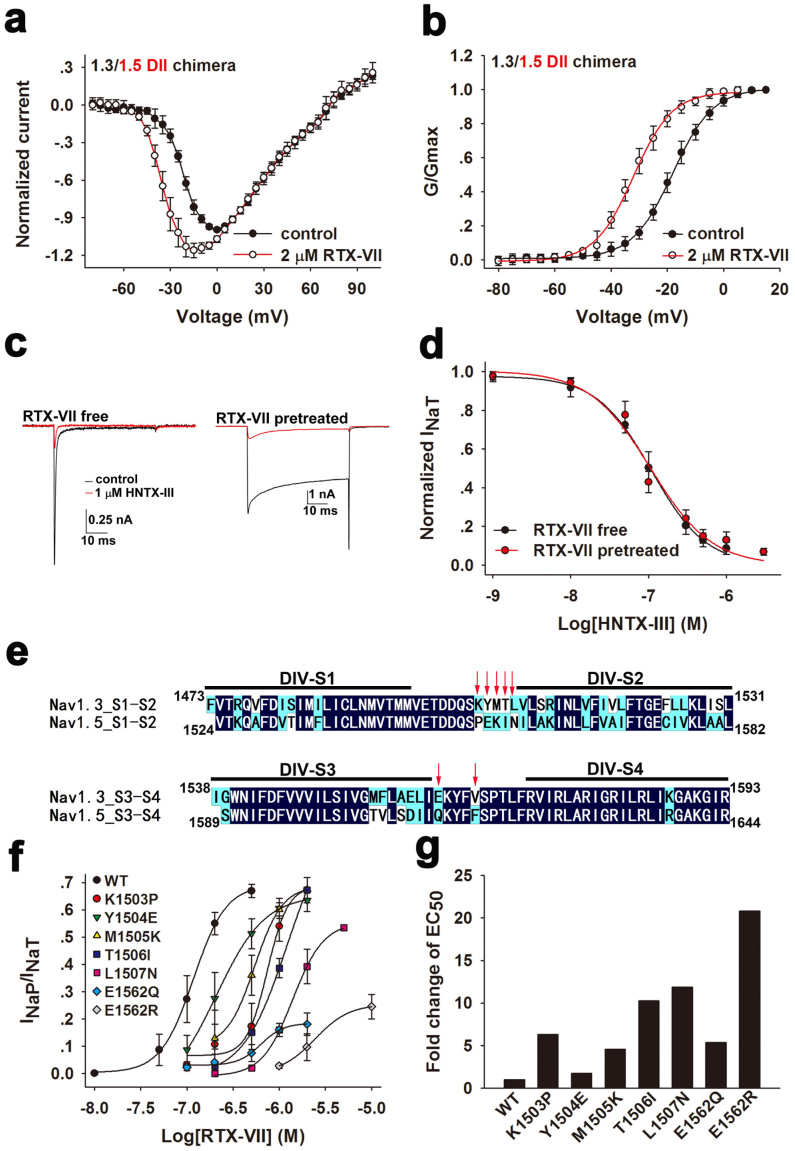
Domain IV but not domain II of Na_v_1.3 is involved in interacting with RTX-VII. (a) The I–V curves of Na_v_1.3/1.5 DII chimera before and after 2 μM RTX-VII treatment (n = 6). (b) 2 μM RTX-VII negatively shifts the G–V curve of Na_v_1.3/1.5 DII chimera without changing the slope factor (*V_a_* = −18.00 ± 1.71 mV for control and *V_a_* = −32.09 ± 1.99 mV for the toxin treatment; *K_a_* = 7.23 ± 1.22 mV for control and *K_a_* = 6.82 ± 1.30 mV for the toxin treatment) (n = 6). (c) Representative traces show 1 μM HNTX-III indiscriminately inhibits the I_NaT_ of 0.5 μM RTX-VII-untreated- (*RTX-VII free*) and -treated- (*RTX-VII pretreated*) Na_v_1.3 channel (n = 5). HNTX-III was dissolved in bath solution containing 0.5 μM RTX-VII. (d) The dose-response curve for HNTX-III inhibiting the I_NaT_ of 0.5 μM RTX-VII-treated or -untreated Na_v_1.3 channel shows that the potency of HNTX-III on both types of channel are the same (n = 5). The fractions which are resistant to the high dose of HNTX-III treatment account for 5.12% and 6.77% of the maximum I_NaT_, respectively. (e) Sequence alignment of the DIV-VSD of Na_v_1.3 and Na_v_1.5, red arrows indicate amino acid residues in Na_v_1.3 which were mutated to their counterpart in Nav1.5. (f) Dose-response curves for RTX-VII enhancing the I_NaP_ of Na_v_1.3 mutants shown in Figure 5e demonstrate molecular determinants in Na_v_1.3 for interacting with RTX-VII (n = 5–8); the apparent EC_50_ values are 117.73 nM, 741.48 nM, 208.83 nM, 541.00 nM, 1209.76 nM, 1399.27 nM, 635.04 nM, 2451, 32 nM for wt-Na_v_1.3, K1503P, Y1504E, M1505K, T1506I, L1507N, E1562Q and E1562R, respectively. (g) Bars show the fold changes of apparent EC_50_ values of RTX-VII for mutants compared with that for wt-Na_v_1.3 channel (n = 5–8).

Further experiments were performed to clarify the roles of Na_v_1.3 DII and DIV. First, we examined whether RTX-VII binds to Na_v_1.3 DII. Neurotoxins acting on DII of Na_v_s often cause a negative or positive shift of the activation kinetics of targeted channels^41^. The substitution of the DII of Na_v_1.3 with that of Na_v_1.5 should affect the RTX-VII-induced negative shift of activation kinetics of Na_v_1.3 if the toxin binds to Na_v_1.3 DII, as RTX-VII did not affect the I–V curve of Na_v_1.5 (Supplementary Fig. S5a). Therefore, the activation kinetics of the chimeric channel Na_v_1.3/1.5 DII was investigated before and after the application of 2 μM RTX-VII. RTX-VII negatively shifted the voltage-dependent activation of the Na_v_1.3/1.5 DII chimera and increased the I_NaT_ at voltages ranging from −50 mV to 5 mV (Figure 5a). In addition, 2 μM RTX-VIII caused an approximate 14 mV negative shift of the G–V curve of the Na_v_1.3/1.5 DII chimera without changing the slope factor (*V_a_* = −18.00 ± 1.71 mV for control and *V_a_* = −32.09 ± 1.99 mV for the toxin treatment; *K_a_* = 7.23 ± 1.22 mV for control and *K_a_* = 6.82 ± 1.30 mV for the toxin treatment; the maximum tolerable voltage error was less than 5 mV, the mean maximum Rs-caused depolarizing voltage error was 2.92 ± 2.10 mV, p < 0.001 when compared to the *Va* shifted amplitude) (Figure 5b).This raises the possibility that the toxin might not interact with Na_v_1.3 DII, which was further confirmed by using a competitive assay. HNTX-III is a tarantula toxin that inhibits the I_NaT_ of Na_v_1.3 and Na_v_1.7. It was found that this toxin targeted DII S3–S4 linker of Nav1.7^42^. The wt-Nav1.5 channel and the Na_v_1.3/1.5 DII chimera were resistant to 1 μM HNTX-III treatment, whereas the Na_v_1.3/1.5 DII-PD chimera was inhibited by 1 μM HNTX-III (Supplementary Fig. S5 b–d). Reconstruction of the DII of Nav1.3 to Nav1.5 (Na_v_ 1.5/1.3 DII chimera) conferred the inhibitory activity of HNTX-III to this channel (Supplementary Fig. S5 e).These evidences indicate HNTX-III inhibit Nav1.3 by binding to its DII-VSD. If RTX-VII also targeted DII-VSD of Na_v_1.3, its binding should prevent the interaction of HNTX-III with Na_v_1.3 because of steric hindrance, which would result in an attenuation of the inhibitory potency of HNTX-III on Na_v_1.3. As shown in Figure 5c, the inhibitory effects of HNTX-III on Na_v_1.3 I_NaT_ did not differ between 0.5 μM RTX-VIII-pretreated and -untreated channels. The dose-response curves were also superimposed well (Figure 5d), providing evidence to rule out the binding of RTX-VII to Na_v_1.3 DII. Next, we determined the molecular determinant in DIV of Na_v_1.3 for RTX-VII binding. Since Na_v_1.5 is resistant to RTX-VII, the residues in S1–S2 and S3–S4 extracellular loops of Na_v_1.3 were mutated to the corresponding residues of Na_v_1.5, respectively (Figure 5e). A total of seven residues were mutated and six of them were functionally expressed except V1566F. The kinetics for the activation and SSI of all mutants were listed in supplementary Table S1. Compared with wt-Na_v_1.3, Four mutant channels (K1503P, M1505K, T1506I, L1507N) carrying mutations in the S1–S2 linker led to a 4–12 folds increase of apparent EC_50_ values, whereas the E1562Q and E1562R mutation in the S3–S4 linker resulted in an approximate 5 folds and 20 folds increase of the apparent EC_50_ values (Figure 5f and 5g). These data indicate that multiple residues located in Na_v_1.3 DIV were involved in interacting with RTX-VII, and that E1562 was the most important residue for the interaction.

### Reverse reconstruction of Na_v_1.3 DII and DIV into Na_v_1.5 fully restores toxin efficacy

**Figure 6:**
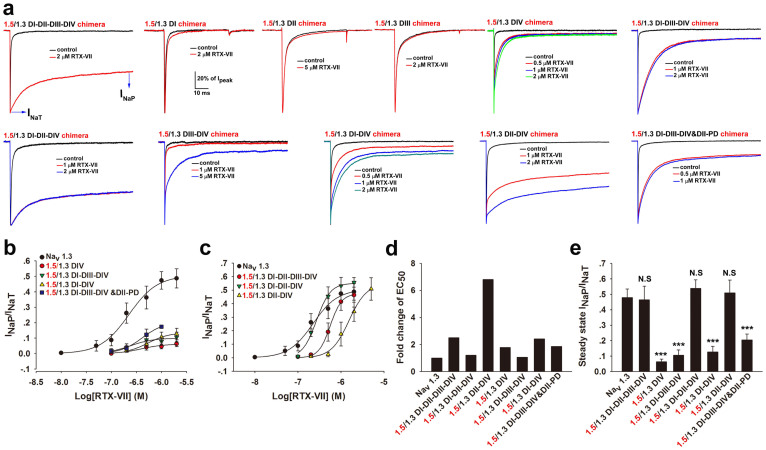
The substitution of the domain II and IV of Na_v_1.5 with those of Na_v_1.3 restores RTX-VII efficacy. (a) A 300-ms recording of the currents of Na_v_1.5 derived chimeric channels in the absence and presence of various concentration of RTX-VII (n = 6–9). The chimeric channels were constructed as follows: one or several domains (DI, DII, DIII or DIV) of Na_v_1.5 were substituted with the corresponding domain/s of Na_v_1.3 (see Supplementary Fig. S6). (b) Dose-response curves for RTX-VII enhancing the I_NaP_ of wt-Na_v_1.3 and Na_v_1.5 derived chimeric channels that did not or slightly restored toxin efficacy (steady-state I_NaP_/I_NaT_ ratio at the saturated concentration of toxin) (n = 6–9). (c) Dose-response curves for RTX-VII enhancing I_NaP_ of wt-Na_v_1.3 and Na_v_1.5 derived chimeric channels that almost completely restored toxin potency and/or efficacy (n = 6–9). (d) Bars show the fold changes of the apparent EC_50_ of RTX-VII for each Na_v_1.5 derived chimeric channels compared with that for wt-Na_v_1.3 (n = 6–9). (e) Bars show the steady- state I_NaP_/I_NaT_ ratio of wt-Na_v_1.3 and Na_v_1.5 derived chimeric channels in the presence of saturated concentrations of toxin. This values are 47.92 ± 5.43%, 46.37 ± 8.87%, 6.3 ± 1.82%, 10.60 ± 3.32%, 53.81 ± 5.56%, 12.68 ± 3.67%, 50.88 ± 8.33 and 20.41 ± 3.90% for Na_v_1.3, 1.5/1.3 DI-DII-DIII-DIV, 1.5/1.3 DIV, 1.5/1.3 DI-DIII-DIV, 1.5/1.3 DI-DII-DIV, 1.5/1.3 DI-DIV, 1.5/1.3 DII-DIV and 1.5/1.3 DI-DIII-DIV&DII-PD, respectively (***p < 0.001, N.S = not significant, when compared with wt-Na_v_1.3) (n = 6–9).

Considering the critical role of the DII and DIV of Na_v_1.3 in the RTX-VII-induced I_NaP_, we assumed that reverse reconstruction of Na_v_1.3 DII and DIV into Na_v_1.5 might restore the efficacy of the toxin. A reversal chimeric strategy was used as follows: four domains of Na_v_1.3 were stepwise reconstructed into the scaffold of Na_v_1.5 (Supplementary Fig. S6). The nomenclature of a chimeric channel was defined as follows: for example, Na_v_1.5/1.3 DI was a chimeric channel in which the DI of Na_v_1.5 was substituted with that of Nav1.3. A total of 11 chimeric channels were constructed and their I_NaP_ generation by the toxin was compared. Again, I_NaP_ was measured at the time point of 295 ms (Figure 6a). The substitution of all four domains of Na_v_1.5 with those of Na_v_1.3 (Nav1.5/1.3 DI-II-III-IV) almost fully restored the efficacy of RTX-VII, thus eliminating the involvements of the intracellular loops of Na_v_1.3 in the toxin-induced I_NaP_. Of the four single domain replaced chimeric channels, Na_v_1.5/1.3 DI, Na_v_1.5/1.3 DII and Na_v_1.5/1.3 DIII chimeras were resistant to RTX-VII, similar to wt-Na_v_1.5, whereas Na_v_1.5/1.3 DIV chimera was sensitive to RTX-VII. Furthermore, the toxin slowed the inactivation and induced a small steady- state I_NaP_ in this chimeric channel, indicating that Na_v_1.3 DIV is important but not sufficient for RTX-VII inducing large I_NaP_. Of the two triple domain replaced chimeric channels, Na_v_1.5/1.3 DI-III-IV chimera did not fully restore toxin efficacy but Na_v_1.5/1.3 DI-II-IV chimera did, which indicates that the DII but not the DI and DIII of Na_v_1.3 is required for toxin inducing large I_NaP_. Of the three double domain replaced chimeric channels, the reconstruction of the DI or DIII of Na_v_1.3 into the scaffold of Na_v_1.5/1.3 DIV chimera (Na_v_1.5/1.3 DIII-IV chimera or Na_v_1.5/1.3 DI-IV chimera) had a limited effect on restoring toxin efficacy, whereas the reconstruction of the DII of Na_v_1.3 into Na_v_1.5/1.3 DIV chimera (Nav1.5/1.3 DII-IV chimera) almost fully rescued toxin efficacy, suggesting the assembly of the DII and DIV of Na_v_1.3 should be sufficient for RTX-VII inducing large I_NaP_. Additionally, the chimeric channel Na_v_1.5/1.3 DI-III-IV&DII PD, where only the DII-PD but not the whole DII of Na_v_1.3 was present, also attenuated the efficacy of RTX-VII compared with that of Na_v_1.5/1.3 DI-II-III-IV chimera, which strongly supports that the DII-VSD of Na_v_1.3 plays a vital role in toxin-induced I_NaP_ generation.

The apparent EC_50_ values of RTX-VII on the Na_v_1.5 derived chimeric channels containing Na_v_1.3 DIV were estimated from the dose-response curves (Figures 6b and c), and the changes in the potency and efficacy of RTX-VII on these chimeric channels were showed in Figures 6d and e, respectively. Of the Na_v_1.3 DIV-containing chimeric channels, the Na_v_1.3 DII-containing ones (Na_v_1.5/1.3 DI-II-III-IV, DI-II-IV and DII-IV chimeras), but not those without reconstruction of Na_v_1.3 DII or DII-VSD (Na_v_1.5/1.3 DIV, DI-III-IV, DI-IV and DI-III-IV&II-PD chimeras), produced a large steady-state I_NaP_ comparable to that of wt-Na_v_1.3 in the presence of saturated concentration toxin (Figure 6e). On the other hand, the toxin potency on these Na_v_1.3 DIV-containing chimeric channels were only slightly weaker than that of wt-Na_v_1.3, although the greatest fold change of EC_50_ was observed in Na_v_1.5/1.3 DII-DIV chimera (8 folds) (Figure 6d). Moreover, the incorporation of Na_v_1.3 DI into Na_v_1.5/1.3 DII-DIV chimera (Na_v_1.5/1.3 DI-DII-DIV chimera) led to an evident enhancement of toxin potency, which is comparable to that of wt-Na_v_1.3 channel. The results are consistent with the interpretation that the DIV of Na_v_1.3 was the main toxin binding site, while the DI-PD of Na_v_1.3 might construct the low affinity binding site for RTX-VII. Overall, combining data in Figures 4 and 6 confirmed the cooperative involvement of DII and DIV in the toxin-induced I_NaP_ of Na_v_1.3.

## Discussion

Neurotoxins produced by venomous animals, plants, and microorganisms are a valuable pool of molecular probes to investigate the structure-function relationship of Na_v_s^43^. RTX-VII robustly enhances the I_NaP_ of Na_v_1.3 and discriminates Na_v_ subtypes Na_v_1.4, Na_v_1.5, and Na_v_1.7–1.9 from Na_v_1.3. The toxin-induced and the intrinsic I_NaP_ share some common features, such as sub-threshold activation, a close correlation with I_ramp2_, and triggering spontaneous high frequency AP firing. Furthermore, the brief late opening and burst of openings of Na_v_s may be the common mechanism underlying the origin of both types of I_NaP_. However, the intrinsic I_NaP_ of Na_v_s is small, which hampered the investigation of the mechanism underlying I_NaP_ generation. RTX-VII dramatically enhancing the I_NaP_ of Na_v_1.3 enabled detailed investigations of Na_v_1.3I_NaP_ generation. In the present study, we clarified the roles of the four domains of Na_v_1.3 in I_NaP_ generation by using RTX-VII as a molecular probe.

Along with the enhancement of I_NaP_, RTX-VII also facilitates Na_v_1.3 channel opening at weak depolarizations as revealed by the toxin potentiating I_NaT_ of Na_v_1.3 when depolarizing voltages are below 10 mV as well as the toxin negatively shifting channel's steady-state activation. This observation is not without precedent, as some α-scorpion toxins modulate Na_v_s in a similar way^37^. This phenomenon could be reasonably interpreted as an increase of the maximum opening probability of the toxin-treated channels. However, how the toxin-bound channels open with a greater probability at weak depolarizations remains unclear. Our data indicate that RTX-VII binds to the DIV-VSD instead of the DII-VSD of Na_v_1.3, which suggests that the potentiation of Na_v_1.3 activation by the toxin might not derive from the toxin facilitating DII activation. Two possible explanations for RTX-VII enhancing the I_NaT_ of Na_v_1.3 are proposed: (1) toxin treatment altered single channel conductance^44^ of Na_v_1.3. This interpretation seems unreasonable because the toxin does not alter the inward and outward Na^+^ current of Na_v_1.3 evoked by strong depolarizing voltages above 10 mV; (2) RTX-VII tends to stabilize the DIV-VSD of Na_v_1.3 in a partially activated state (pre-activated state), which is required for channel activation but not sufficient to trigger channel inactivation (the fully activated DIV-VSD is required for the fast inactivation of Na_v_s)^45^, thus promoting channel activation in a similar but not identical way with that of β-scorpion toxins^46^,^47^. The vital difference is that β-scorpion toxins trap the DII-VSD but not DIV-VSD of Na_v_s in the activated state. The latter interpretation seems plausible as emerging evidences support DIV is involved in Na_v_ activation^45^,^48^. Furthermore, the voltage driving the outward movement of DIV-VSD is the later step in the activation sequence of Na_v_s^49^. RTX-VII did not alter the I_NaT_ of Na_v_1.3 at strong depolarizing voltages (>10 mV), and this phenomenon could be interpreted by the fact that both toxin-free and toxin-bound channels in cell membrane are almost fully activated at 10 mV, which is in consistent with the G-V relationship observed in figure 2d. Taken in all, considering RTX-VII promoting activation and inhibiting inactivation of Na_v_1.3 as well as the unique role of DIV-VSD in channel gating, we would like to suggest that RTX-VII might tend to trap and stabilize the DIV-VSD of Na_v_1.3 in the pre-activated state during channel activation.

The reconstruction of Na_v_1.3 DII but not DI or DIII to Na_v_1.5/1.3 DIV chimera fully restored toxin efficacy, but it is interesting that RTX-VII did not bind to Na_v_1.3 DII. Therefore, the role of this domain in the toxin-induced I_NaP_ remains unclear. Previous studies showed that the inter-domain interactions of Na_v_s is necessary for channel gating^50^,^51^. We proposed in this study that the DII and DIV of Na_v_1.3 might cooperate to trigger late brief opening and burst of opening to generate I_NaP_, and RTX-VII should facilitate/amplify this cooperation to induce large I_NaP_ in Na_v_1.3. The subtle amino acid sequence differences of the domain II between Na_v_1.3 and Na_v_1.5 greatly affect this cooperation, namely the DII of Na_v_1.3 can cooperate well with its own DIV, which is not the case for the DII of Na_v_1.5 with DIV of Na_v_1.3. The roles of Na_v_1.3 DI and DIII of in the toxin-induced I_NaP_ generation were unclear. The fact that the replacement of the DI or DIII of Na_v_1.3 with that of Na_v_1.5 did not affect toxin efficacy could not exclude the possibility that both domains might involve in the I_NaP_ generation, because high sequence similarity of DI and DIII between Na_v_1.3 and Na_v_1.5 is observed and probably the inter-domain interactions might not be interfered although these two domains were replaced.

RTX-VII induced large I_NaP_ in Na_v_1.3 at the end of a 50-ms or a 300-ms depolarization, which differs from some scorpion toxins and sea anemone toxins that slow the inactivation of Na_v_s but the resultant current decay rapidly in 50 ms (Lqh2 as a representative^52^). What is the difference derived from? Theoretically, Lqh2 trapping the DIV-VSD of Na_v_s in the closed state should have induced large I_NaP_, but the fact is not. How are the toxin-bound channels inactivated? Slow inactivation may not be the underling mechanism. This is because that slow inactivation is rarely observed in a 50 ms depolarization (such a short depolarization is not sufficient to trigger this gating process). The repriming kinetics of the toxin-bound channels is the same as or even faster than that of the toxin-free channels^37^, which is also inconsistent with the fact that the recovery of Na_v_s from slow inactivation is slow^53^. Based on the unique role of DIV in fast inactivation, a model was proposed to clarify these two problems. Macroscopically, in this model, a depolarization would drive and maintain the first three domains of Na_v_s in an activated state; Lqh2 and RTX-VII could trap the DIV-VSD of Na_v_s in the closed^52^ and partially activated state, respectively. For Lqh2, such trapping is not very stable, as the depolarization prolongs, the toxin-bound DIV-VSD would be gradually activated, triggering channel inactivation. However, for RTX-VII, the DII of Na_v_1.3 might allosterically slow/inhibit this process, which therefore makes RTX-VII stably trap the DIV-VSD of Na_v_1.3 in the partially activated state and then the channels would maintain a persistent opening state. The understanding of this process in the single channel level could be as follows: the inactivation ball of a Na_v_ has a “on state” (blocking the pore) and an “off state” (free in cytosol) which are tightly coupled to the activated and resting state of DIV-VSD, respectively^8^. Normally, DIV-VSD is immobilized in an outward conformation by activation^54^,^55^. The toxin-bound DIV-VSD could be activated by strong depolarization but not be stably immobilized as toxins tend to “drag” the DIV-VSD to its resting state (partially activated state for RTX-VII). Thus, when the toxin-bound DIV-VSD is activated, the inactivation ball is in the “on state” and the pore is occluded; when the toxin-bound DIV-VSD is in the resting state (partially activated state for RTX-VII), the channel just opens. The inactivation ball switches between the “on state” and the “off state” quickly and such inactivation ball movement should trigger the burst opening of the channel in single channel recording. For Lqh2, as the depolarization prolonged, the DIV-VSD of most channels would be stably immobilized and these channels were consequently trapped stably in the inactivated state. On the other hand, for RTX-VII, toxin binding to the DIV-VSD of Na_v_1.3 should allosterically affect the conformation of DII-VSD, which would in turn interfere with the time-dependent immobilization of DIV-VSD. We proposed that such gating model underlies the generation of large I_NaP_ in Na_v_1.3 by RTX-VII.

## Methods

### Venom and toxin purification

Spider *Macrothele raveni* were collected in GuangXi province, China. The spider has a body length of 3–5 cm and the venom was collected by an electric stimulation method as described in another work of our laboratory^34^. The collected crude venom was lyophilized and preserved at −80°C before use. The crude venom was dissolved in ddH_2_O to a final concentration of 5 mg/ml and subjected to the first round of RP-HPLC purification (acetonitrile gradient: 1%–60%, at an increasing rate of 1% per minute). The fraction containing RTX-VII was then collected, lyophilized and subjected to the second round of RP-HPLC with a slower increasing acetonitrile gradient (acetonitrile at an increasing rate of 0.5% per minute) to obtain the purified toxin.

### Toxin sequencing and cDNA of RTX-VII

Partial amino acid sequence of RTX-VII was determined by Edman degradation on an Applied Biosystems/PerkinElmer Life Science Procise 491-A protein sequencer. The cDNA of this toxin was obtained by blasting Edman degradation determined amino acid sequence of RTX-VII against the local cDNA library database of the spider *Macrothele raveni* (unpublished data).

### Constructs and transfection

All Na_v_ clones and beta subunit clones were kindly gift from Dr Theodore R.Cummins (Department of pharmacology and Toxicology, Stark Neurosciences Research Institute, Indiana University School of Medicine, USA). cDNA genes encoding rat Na_v_1.3 and rat Na_v_1.4 were subcloned into the vectors pcDNA3.1 and pRGB4^40^,^56^, respectively; the cDNA genes encoding human Na_v_1.5 and human Na_v_1.7 were subcloned into the vectors pcDNA3.1 and pcDNA3.1-mod^57^, respectively. Auxiliary β1 and β2 subunits both were cloned from human and inserted into an internal ribosome entry site vector^58^. All site mutations of Na_v_1.3 were constructed by using the QuikChange II XL Site-directed Mutagenesis kit (Agilent Technologies) according to the manufacture's instruction. The cytosolic boundaries of two adjacent transmembrane segments and two adjacent domains of Na_v_1.3 or Na_v_1.5 were determined by proteins' topological information deposited in NCBI protein database (for Na_v_1.3, the website is http://www.ncbi.nlm.nih.gov/protein/NP_037251.1, and for Na_v_1.5,the website link is http://www.ncbi.nlm.nih.gov/protein/NP_932173.1). The protein sequence location of each voltage sensor (VSD)/pore domain (PD) of all four domains of Na_v_1.3 and Na_v_1.5 are as listed in Supplementary Table S4. A homologous recombination strategy was employed to generate the chimeric channels using the In-FusionHD Cloning kit (Clontech Laboratories) or CloneEZ PCR Cloning kit (Genscript). For example, for the construction of Na_v_1.3/1.5 DI-VSD chimera, the DI-VSD (voltage sensor of domain I) of Na_v_1.5 was amplified by PCR using a pair of primers with their 5′ end extended by a 15 bp long joint which is homologous or reverse compliment to the upstream or downstream flanking sequence of DI-VSD of Na_v_1.3. A pair of oppositely directed primers was used to linearize the whole Na_v_1.3 cloned plasmid with the DI-VSD of Na_v_1.3 deleted. The PCR amplified segment and the linearized plasmid were subjected to 1% agarose gel electrophoresis, respectively. The corresponding bands were recycled using a DNA gel extraction kit (Sangon biotech) and ligated using the In-FusionHD Cloning kit (Clontech Laboratories) or CloneEZ PCR Cloning kit (Genscript). Before being transformed to E.coli Top10 competent cell, the ligated product was subjected to FastDigest DpnI (Thermo Scientific) treatment at 37°C for 1 hour to remove the template plasmid. The transformants were verified by colony-PCR using a pair of gene specific primer for each inserted segment and then sequencing (Genscript). The primers used for vector linearization and amplification of Na_v_ domains were listed in Supplementary Table S2 and Table S3. HEK293T cells (ATCC) were grown under the standard cell culture conditions (5% CO_2_ and 37°C) in Dulbecco's Modified Eagle Medium (DMEM, Life technologies) supplemented with 10% fetal bovine serum. These Na_v_ constructs were co-transfected with plasmid containing β1 subunit and PEGFP-N1 to HEK293T cells using Lipofectamine 2000 (Life Technology) according to the manufacture's instruction. For wt-Na_v_1.3, Na_v_1.3 mutants and Na_v_1.3 derived chimeric channels, 3 μg Na_v_ plasmid, 1 μg plasmid containing β1 subunit and 0.5 μg PEGFP-N1 plasmid were co-transfected. For wt-Na_v_1.5 and Na_v_1.5 derived chimeric channels, 1 μg Na_v_ plasmid, 0.3 μg plasmid containing β1 subunit and 0.5 μg PEGFP-N1 plasmid were co-transfected. For ramp test, Na_v_1.3 was co-transfected with plasmid containing β1 subunit and plasmid containing β2 subunit^33^. Cells were 80%–90% confluent before transfection, and cells were seeded on a poly-lysine coated Microscope Cover Glass (Fisher scientific) 4–6 hours after transfection. 24 hours after seeding, cells were ready for patch-clamp analysis.

### Primary culture of DRG and hippocampal neurons and toxicity test of animals

Animals (Sprague-Dawley rats and Kunming mice) were used according to the guidelines of the National Institutes of Health for care and use of laboratory Animals. The experiments were approved by the Animal Care and Use Committee of the College of Medicine, Hunan Normal University. Acutely dissociated dorsal root ganglion (DRG) cells were prepared from 4 weeks old Sprague-Dawley rats and maintained in short-term primary culture using the method described by Hu, H.Z and Li, Z.W^59^. The dissociated cells were suspended in DMEM supplemented with 10% fetal bovine serum, 50 IU/ml penicillin, and 50 μg/ml streptomycin. Cells were seeded on poly-L-lysine-coated Microscope Cover Glass placed in a cell culture dish (35 × 10 mm, corning) and incubated at 37°C in an atmosphere of 5% CO_2_. Cells cultured for 3–24 h were used in the patch experiments. Experiments were conducted at room temperature (20–25°C). For primary culture of hippocampal neurons, hippocampal tissues of neonatal rats were dissected and treated with 0.25% trypsin in Ca^2+^-Mg^2+^-free Hank's Buffered Salt solution at 37°C for 15 min, and then were dissociated by trituration with glass Pasteur pipette and seeded on poly-L-lysine-coated Microscope Cover Glass placed in a cell culture dish (35 × 10 mm, corning). Approximate 3.5*10^4^ cells in DMEM containing 10% fetal bovine serum were plated in each dish. The culture medium were replaced with serum-free Neurobasal medium (Life technologies) supplemented by 2% B27 (Life technologies) on the second day after plating, 500 μM glutamine was added to reduce the growth of glial cells. The hippocampal neurons were maintained in a CO_2_ incubator at 37°C, one-half volume of the culture medium was replaced with fresh medium every other day. The neurons were used for patch-clamp analysis after they were maintained in culture for 14–17 days. In order to test the neurotoxicity of RTX-VII, ten mice of either sex with an average weight of 20 g were randomly divided to two groups, animals in the control group were intracerebroventricularlly injected with 20 μL saline, and animals in the experimental group were injected with 20 μL saline containing 20 ng toxin.

### Electrophysiology

Cell current recording was made with the whole-cell patch-clamp technique using an EPC 10 USB Patch Clamp Amplifier (HEKA Elektronik). Cells transfected with wt/mutant/chimeric Na_v_ channels and DRG/hippocampal neurons seeding in a glass coverslip were placed in a perfusion chamber in which rapid exchange of solutions around cells could be performed. The recording pipettes were made from glass capillary (thickness = 0.225 mm) using a PC-10 puller (NARISHIGE).The pipet resistance was controlled at 1.5–2.0 MΩ by adjusting the pulling temperature. The standard pipet solution contained (in mM): 140 CsCl, 10 NaCl, 1 EGTA, 2 Mg-ATP, and 20 HEPES (pH 7.4). Bath solution contained (in mM): 140 NaCl, 2 CaCl_2_, 1 MgCl_2_, 5 KCl, 20 HEPES (pH 7.4), and 10 glucose. All experiments were conducted at the room temperature (20–25°C). All chemicals were the products of SigmaAldrich and dissolved in water. Data were acquired by PatchMaster software (HEKA Elektronik). Data were analyzed by softwares Igo Pro 6.10A, Excel 2010, Sigmaplot 10.0 and OriginPro 8. Voltage errors were minimized by using 80% series resistance compensation, the speed value of Rs compensation was set to be 10 μs(fast compensation).The capacitance artifact were canceled using the computer-controlled circuitry of the patch clamp amplifier. The pipet capacitance was minimized by filling the pipet with small volume of pipet solution, and the pipet capacitance was controlled to be <10 pF for effective automatic compensation by EPC-10 amplifier. The pipet capacitance and the cell capacitance was sequentially compensated after the seal and the whole-cell configuration was established, respectively(pipet capacitance and cell capacitance was compensated by automatic fast and slow capacitance compensation, respectively).Stock solution of RTX-VII (1 mM in sterile ddH_2_O) was diluted with fresh bath solution to a concentration of 10 folds of the interested concentration, 30 μL of the concentrated toxin was diluted into the recording chamber (containing 270 μL bath solution) far from the recording pipet (the recording cell) and was mixed by repeatedly pipetting to achieve the specified final concentration^60^. The dose-response curves of toxin on wt/mutant/chimeric channel were fitted to a Hill equation to estimate the potency of toxin (EC_50_). The G-V curve before and after toxin treatment and the steady state inactivation (SSI) curve before toxin treatment were fitted using a boltzmann equation: y = 1/(1 + exp[(V_1/2_ − V)/K]) in which V_1/2_, V, and K represented midpoint voltage of kinetics, test potential and slope factor, respectively. The SSI curve after toxin treatment was fitted with a modified Boltzmann equation: (Y − Y_min_)/(Y_max_ − Y_min_) = 1/(1 + exp[(V_1/2_ − V)/K)]), Y_max_ and Y_min_ represent the maximum and minimum responses. The time course curve for the I_NaP_ enhancement in response to the toxin application was best fitted by a single exponential rising equation (y = y_0_ + a(1 − e^−x/τ^)) and the time course for recovery of I_NaP_ upon bath solution washing was best fitted by a single exponential decay equation(y = y_0_ + ae^−x/τ^), here τ represent the time constant for toxin binding to and washing off from channels respectively. In measuring the spontaneous AP firing of neonatal rat hippocampal neuron using current-clamp, the pipette solution contains (in mM): 140 KCl, 5 MgCl_2_, 5 EGTA, 2.5 CaCl_2_, 4 ATP, 0.3 GTP, and 10 Hepes, pH 7.3 (adjusted with KOH). The bath solution contains (in mM): 140 NaCl, 1 MgCl_2_, 5 KCl, 2 CaCl_2_, 10 HEPES, and 10 glucose, pH 7.3 (adjusted with NaOH). During the recording, no current was injected to neurons.

### Data analysis

Data were presented as Mean ± SD. n is presented as the number of the separate experimental cells. Dose response curves were fitted using the following Hill logistic equation: y = f_max_ − (f_max_ − f_min_)/(1+([Tx]/EC_50_)^n^), where f_max_ and f_min_ represent the maximum and minimum response of channel to toxin, [Tx] represent the toxin concentration, n is an empirical Hill coefficient. The Hill coefficient was set to 1 except where indicated otherwise. This is reasonable based on our mutagenesis analysis, which indicated a single high affinity binding site in Na_v_1.3 for RTX-VII. Statistical significance was assessed with Microsoft excel 2010 using One-Way ANOVA. Statistical significance was accepted at *P* values less than 0.05.

## Author Contributions

C.T. and Z.L. designed experiments; C.T., X.Z., Y.Z., Z.X., Y.H., B.C., Z.H. and C.Z. performed experiments; C.T. constructed mutant and chimeric channels, C.T. and X.Z. conducted patch clamp analysis; C.T., Z.L. and S.L. contributed to manuscript preparation; C.T. and Z.L. wrote the manuscript.

## Supplementary Material

Supplementary Information supplementary information

Supplementary Information Supplementary Video 1 - Control Group

Supplementary Information Supplementary Video 2 - Experimental Group
