# Supplementary material for: Synergetic Action of Domain II and IV Underlies Persistent Current Generation in Nav1.3 as revealed by a tarantula toxin
Source: Sci Rep. 2015 Mar 18;5:9241. doi: 10.1038/srep09241 (PMC4363837; doi:10.1038/srep09241)
Supplement: Supplementary Information [file srep09241-s1.pdf]

Synergetic Action of Domain II and IV Underlies Persistent Current Generation in $Na_v1.3$ as revealed by a tarantula toxin

Cheng Tang[1,#], Xi Zhou[1,#], Yunxiao Zhang[1], Zhaohua xiao[2] , Zhaotun Hu[1], Changxin Zhang[1],Ying Huang[1], Bo Chen[1], Zhonghua Liu[1,*], Songping Liang[1,*]

[1]College of Life Science, Hunan Normal University, Changsha，Hunan, 410081, China

[2]Department of Pediatrics, Xiangya Hospital of Central South University, Changsha, Hunan, 410008, China

[*]Corresponding author: Zhonghua Liu, liuzh@hunnu.edu.cn; Songping Liang, liangsp@hunnu.edu.cn

[#]Contribute equally to this study

Supplementary results:

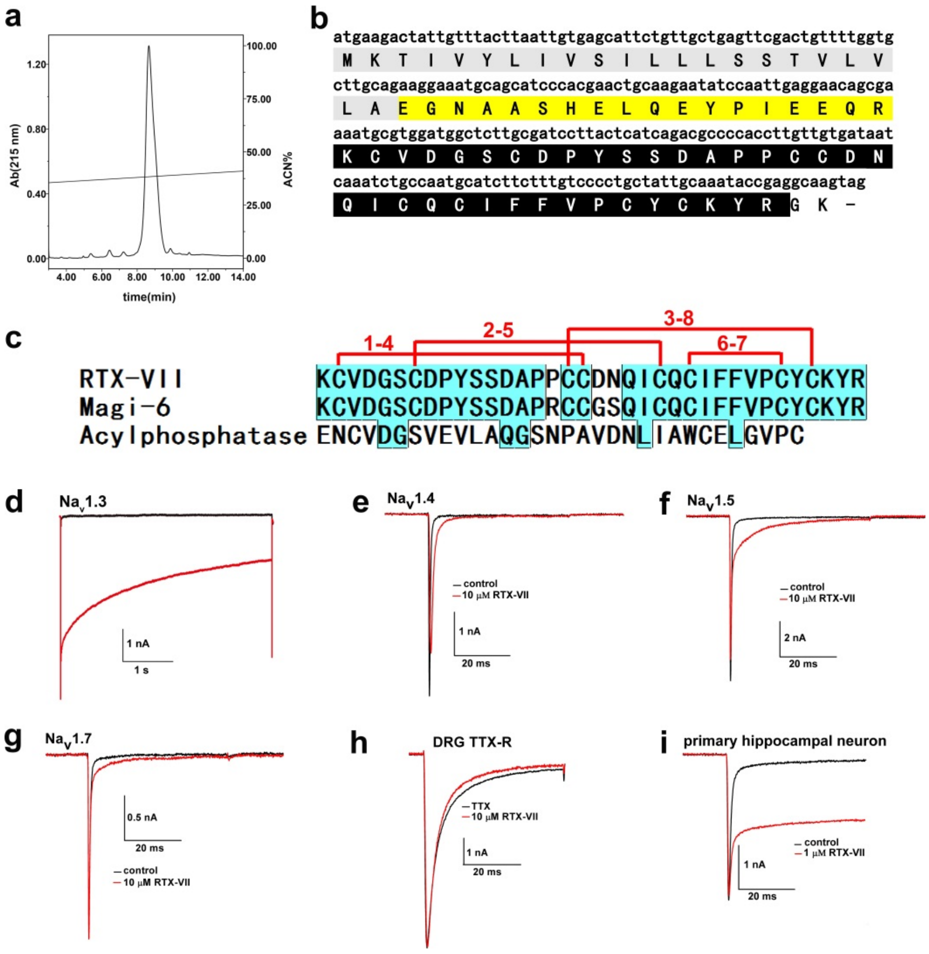

Supplementary Fig S1: (a) RTX-VII was further purified by analytical C_{18} RP-HPLC using a narrower acetonitrile gradient. (b) The cDNA sequence of RTX-VII. The signal peptide is shown in gray, the pro-peptide is shown in yellow and the mature peptide is shown with white text on a black ground. Note partial sequence of the mature peptide was also determined by Edman degradation as shown in supplementary Fig S1j. (c) RTX-VII shows high sequence similarity to the known toxin Magi-6; the possible disulfide mode was also shown. (d) RTX-VII induced large I_{NaP} in $Na_v1.3$ even lasting for several seconds (n = 4). (e, f, g, h) Representative traces showing that $Na_v1.4$, $Na_v1.5$, $Na_v1.7$ as well as DRG TTX-R Na_vs ($Na_v1.8$ and $Na_v1.9$) were resistant to RTX-VII even at the toxin concentration of 10 μM (n = 4 - 6). (i) Representative trace showing Na_vs in neonatal rat hippocampal neurons were highly sensitive to RTX-VII, with 1 μM toxin dramatically inhibiting the inactivation and inducing a large I_{NaP} (n = 6).

Supplementary Fig S1j:

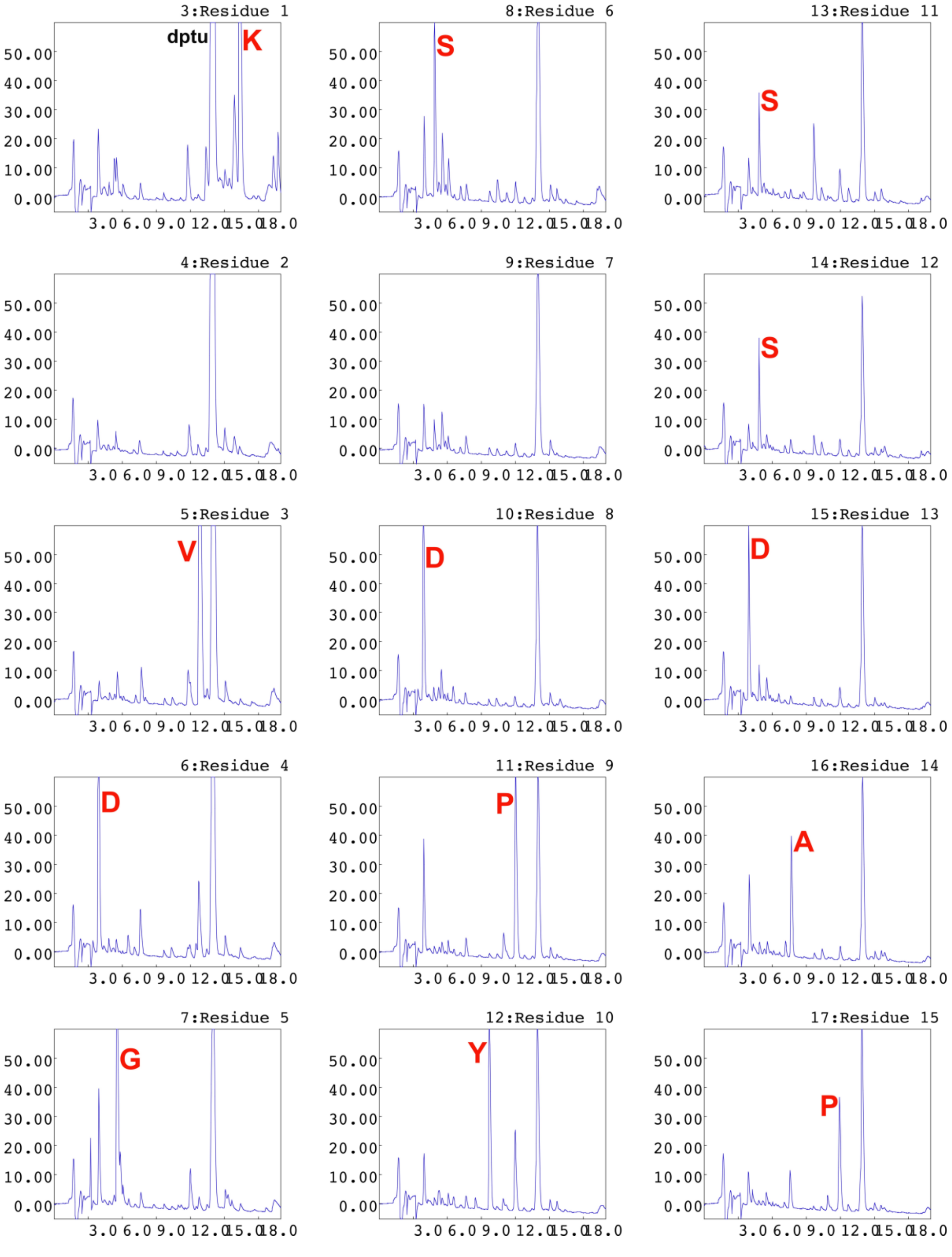

Supplementary Fig S1j(continued):

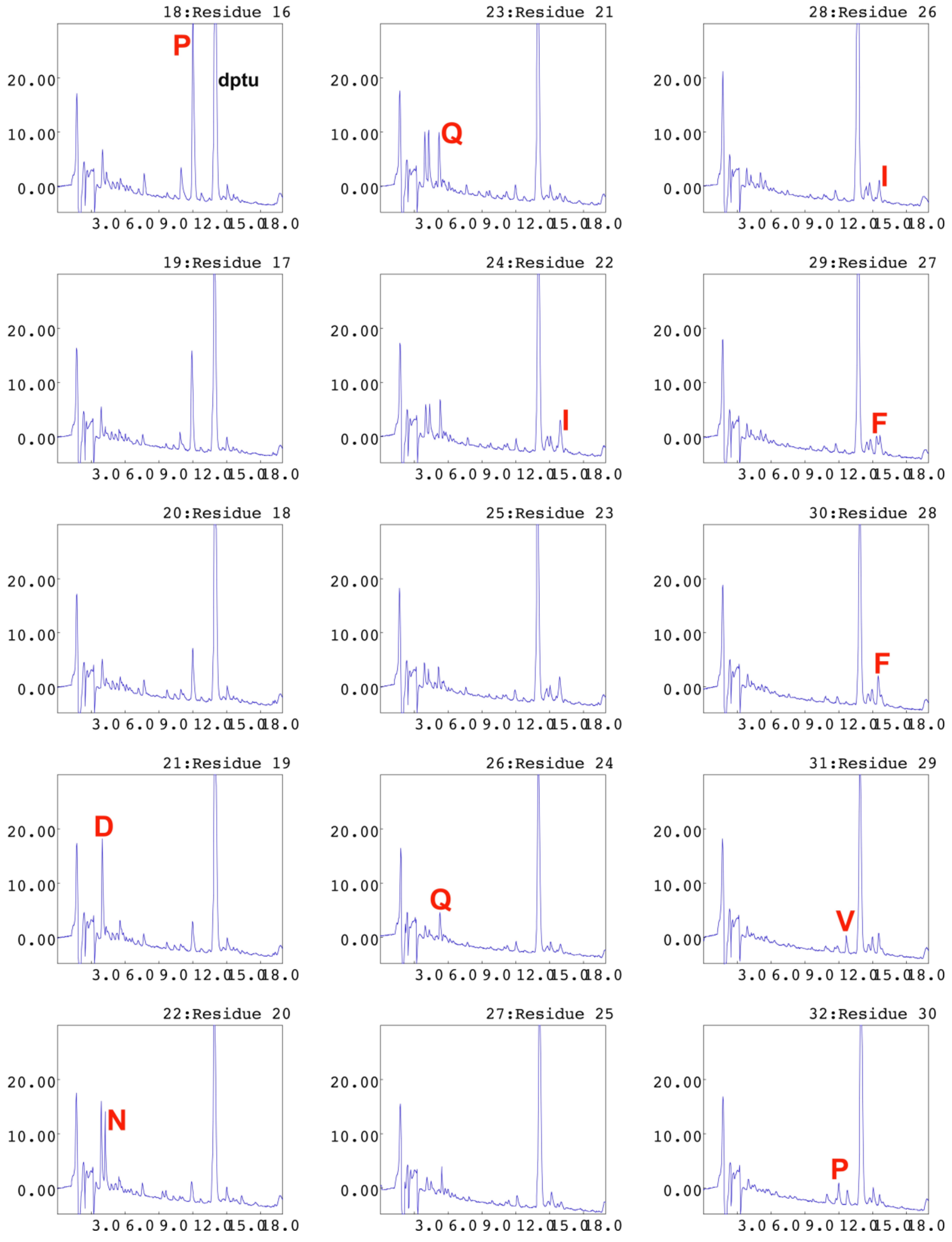

Supplementary Fig S1j: Edman degradation of RTX-VII. Peaks representing the first 30 N-terminal residues could be recognized in the RP-HPLC chromatograms, while the first 22 residues were well characterized. In each cycle, the peak representing the corresponding residue was labeled, and the unlabeled cycle indicated a cysteine residue.

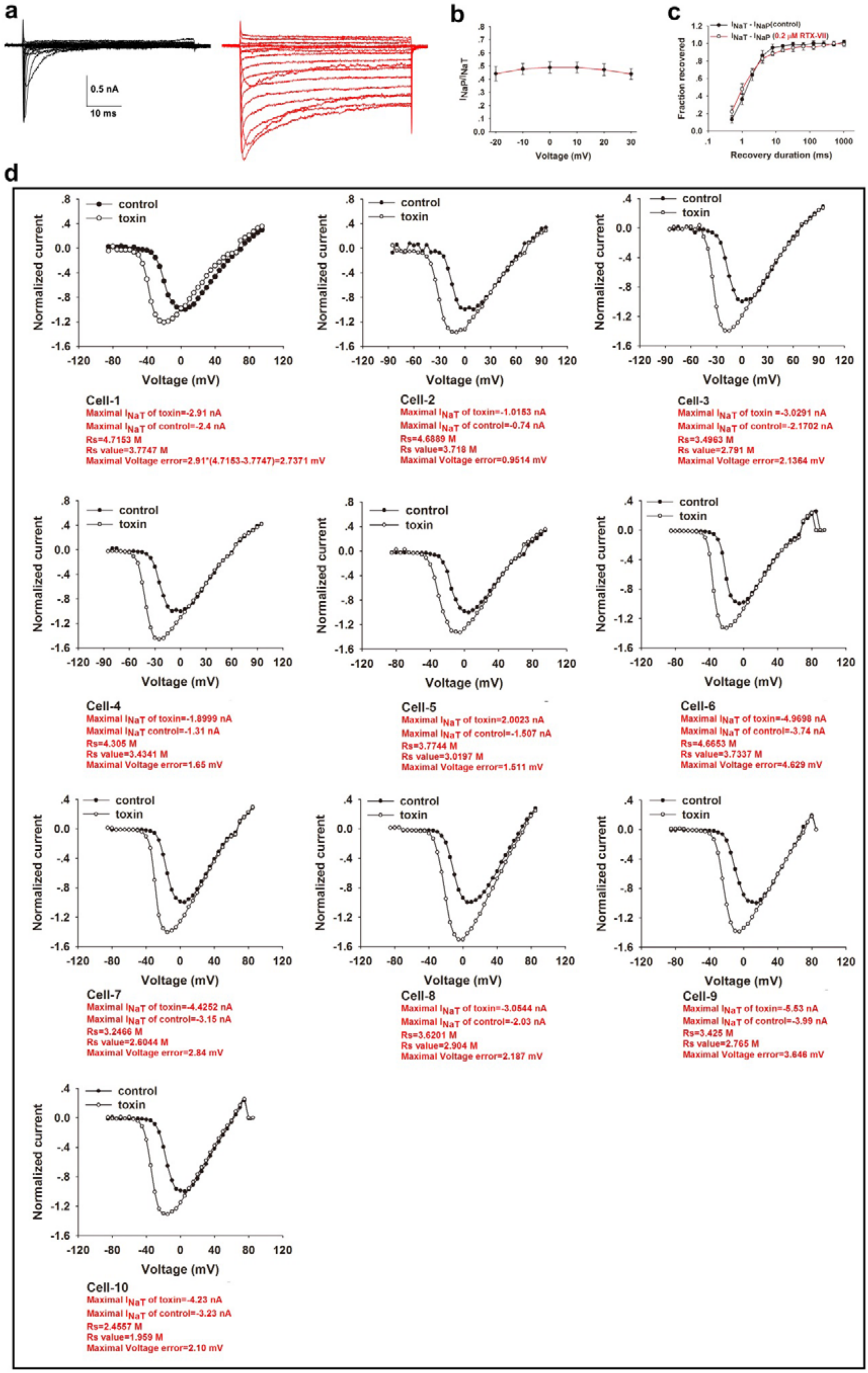

Supplementary Fig S2: (a) A cluster of current traces of $Na_v1.3$ elicited by an I-V protocol

shows 0.2 μM RTX-VII enhanced the inward I_{NaT} but not the outward I_{NaT} and induced large I_{Nap} (*right, red traces*) compared to control (*left, black traces*) (n = 11). (b) 0.2 μM RTX-VII enhanced the I_{NaP} of $Na_v1.3$ in a voltage-independent manner at depolarizing voltages ranging from -20 mV to 30 mV (n = 6). (c) If I_{NaP} was subtracted from I_{NaT} in both control and toxin-treated $Na_v1.3$, the residual transient-inward currents in both groups shared rather similar repriming kinetics (n = 5). (d) Representative I-V traces of ten separate cells expressing $Na_v1.3$ before and after the application of RTX-VII, the current amplitude, the amplitude of uncompensated series resistance, and the maximum uncompensated Rs caused voltage error of each cell were shown.

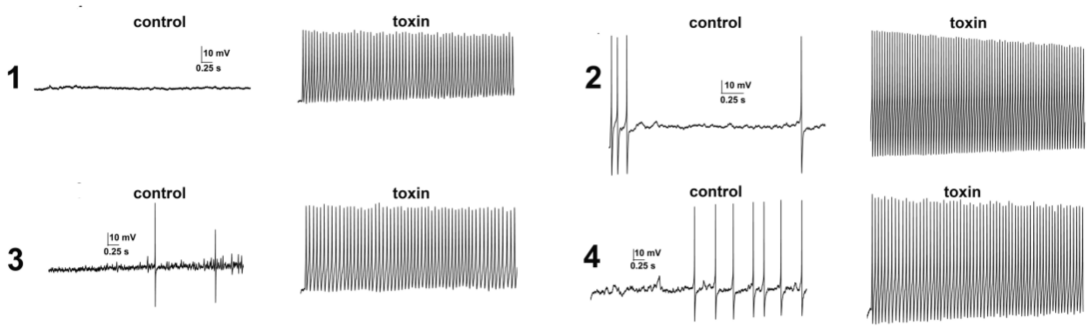

Supplementary Fig S3: Representative traces show RTX-VII triggering spontaneous high frequency AP firing in neonatal rat hippocampal neurons. The data of four cells were shown.

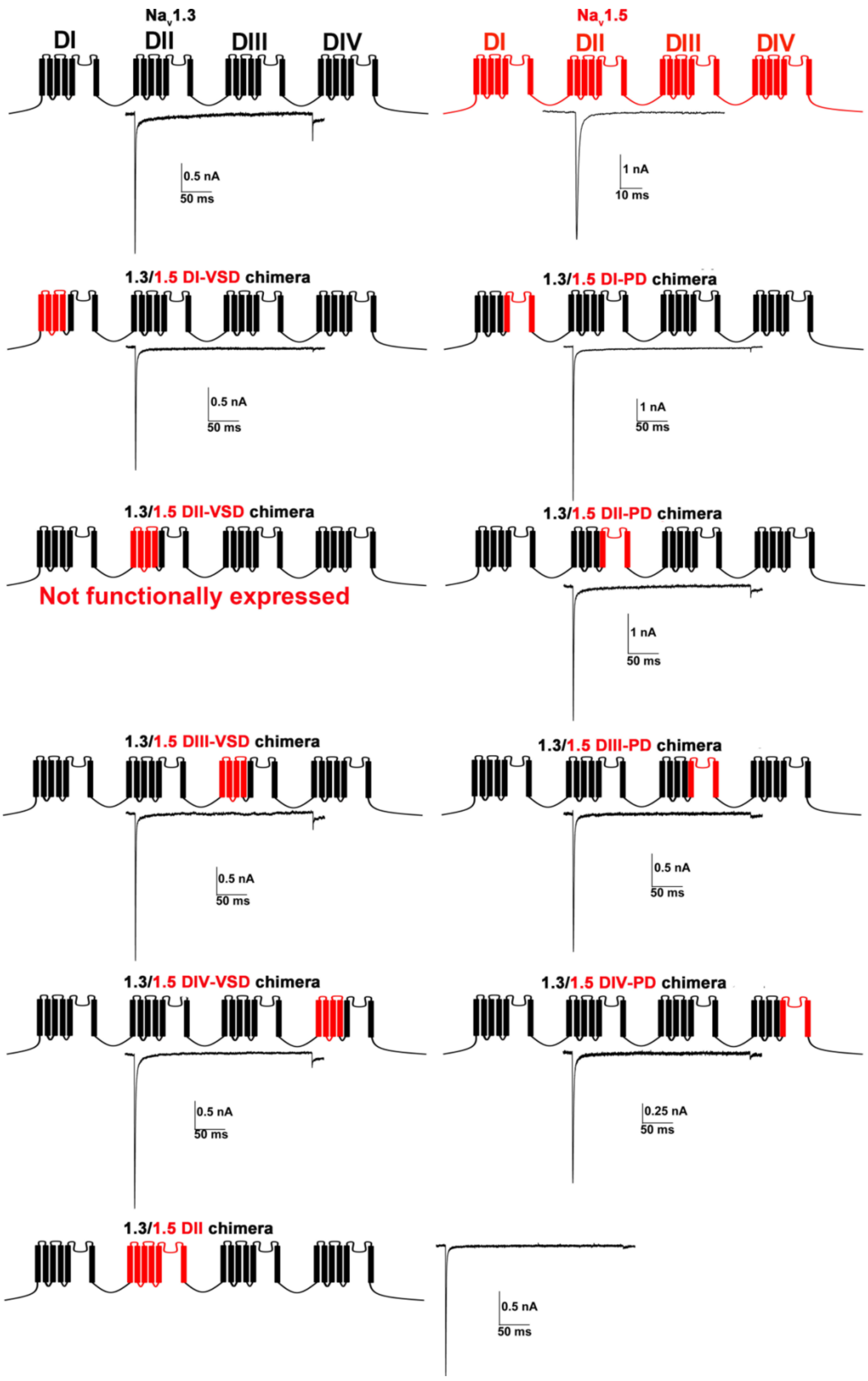

Supplementary Fig S4: Diagrams show the strategy for the construction of Na_v1.3 derived chimeric channels. Na_v1.3 and Na_v1.5 are shown in black and red, respectively. The specific VSD (S1-S4) or PD (S5-S6) in Na_v1.3 was substituted with the corresponding one of Na_v1.5. Except Na_v1.3/1.5 DII-VSD chimera, all the other hybrid channels expressed in HEK293T cells produce large currents in response to a 10 mV depolarization from the holding potential of -100 mV (n = 7-11).

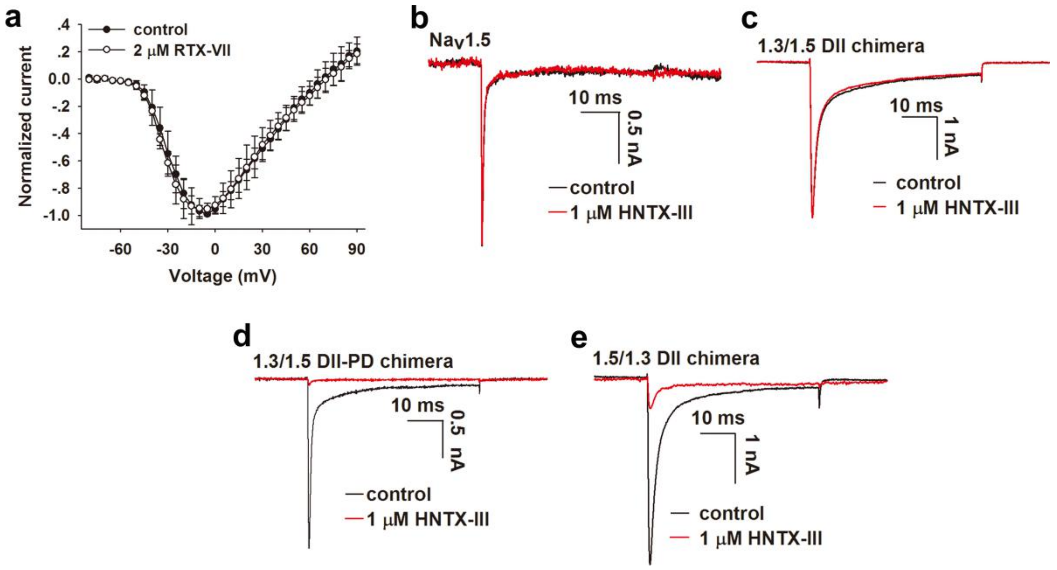

Supplementary Fig S5: (a) RTX-VII did not alter the I-V relationship of Na_v1.5. (b, c, d) Na_v1.5 and 1.3/1.5 DII chimera were resistant to HNTX-III, but the substitution of DII-PD with that of Na_v1.5 (1.3/1.5 DII-PD chimera) did not affect the inhibitory effect of HNTX-III on Nav1.3. (e) The reconstruction of the DII of Na_v1.3 to Na_v1.5 restored the inhibitory activity of HNTX-III.

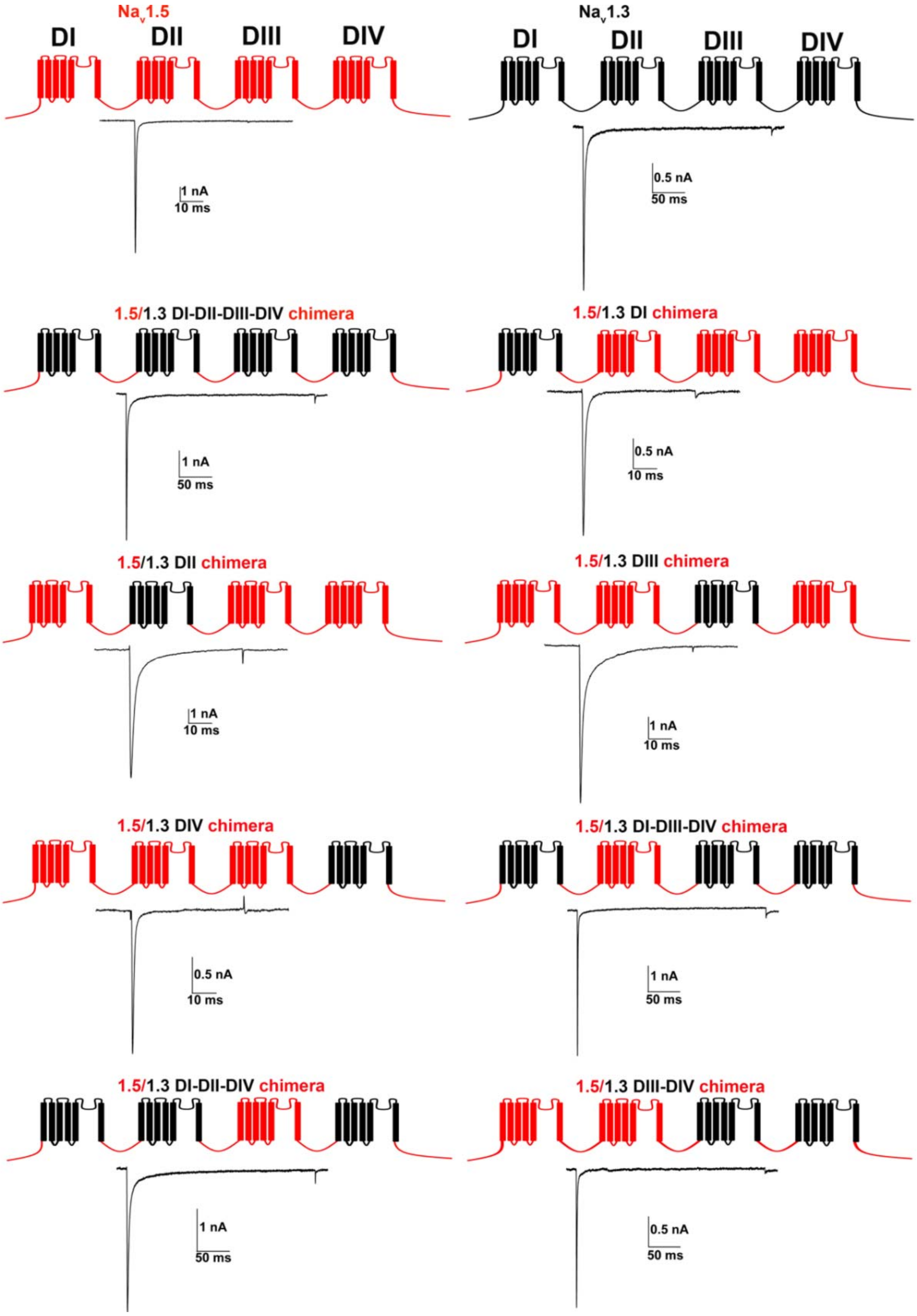

Na$_v$1.5
DI
DII
DIII
DIV
1 nA
10 ms
Na$_v$1.3
DI
DII
DIII
DIV
0.5 nA
50 ms
1.5/1.3 DI-DII-DIII-DIV chimera
1 nA
50 ms
1.5/1.3 DI chimera
0.5 nA
10 ms
1.5/1.3 DII chimera
1 nA
10 ms
1.5/1.3 DIII chimera
1 nA
10 ms
1.5/1.3 DIV chimera
0.5 nA
10 ms
1.5/1.3 DI-DIII-DIV chimera
1 nA
50 ms
1.5/1.3 DI-DII-DIV chimera
1 nA
50 ms
1.5/1.3 DIII-DIV chimera
0.5 nA
50 ms

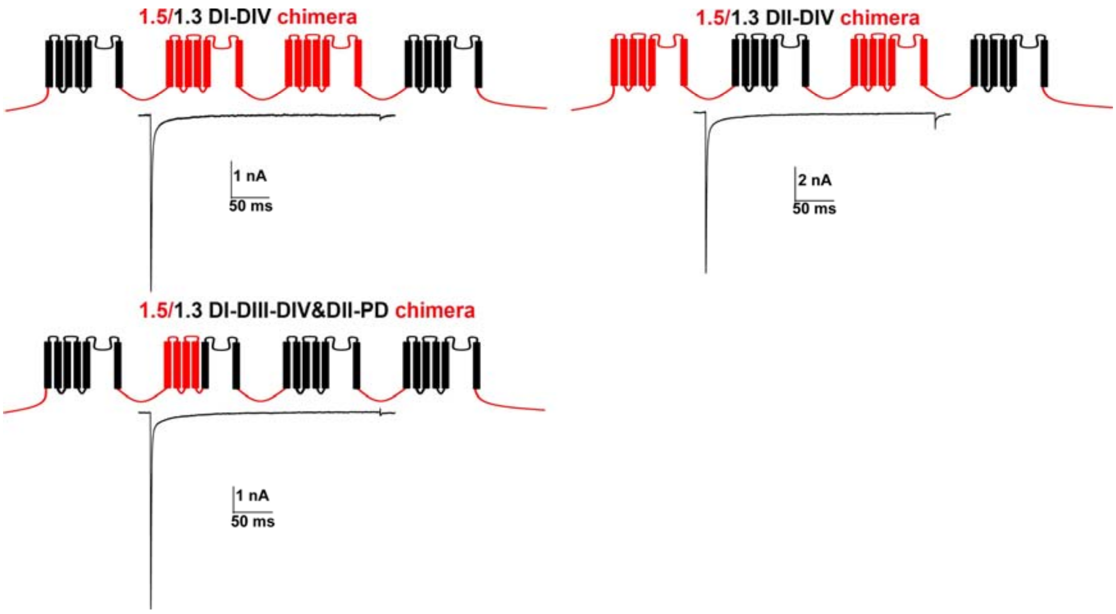

Supplementary Fig S6: Diagrams show the strategy for the construction of Na_v1.5 derived chimeric channels. Na_v1.3 and Na_v1.5 are shown in black and red, respectively. One or several domains of Na_v1.5 were substituted with the corresponding ones of Na_v1.3. All the Nav1.5 derived hybrid channels expressed in HEK293T cells produce large currents in response to a 10 mV depolarization from the holding potential of -100 mV (n = 6 - 9).

Supplementary Table S1: Site mutations in Nav1.3 differently affect channel's steady-state activation and inactivation

	Steady state Activation		Steady state inactivation	
	Va(mV)	Ka(mV)	Vh(mV)	Kh(mV)
WT Nav1.3	-12.71±4.92	6.84±0.98	-44.58±2.73	-6.98±1.44
K1503P	-12.04±3.88	7.62±1.63	-40.38±8.76	-6.51±1.36
Y1504E	-12.43±1.51	7.21±0.79	-43.19±1.09	-6.69±1.50
M1505K	-15.02±3.21	4.66±1.35[c]	-37.36±7.77[a]	-6.01±0.88
T1506I	-10.96±3.45	6.22±0.47	-42.38±7.11	-5.47±0.48
L1507N	-5.30±3.65[b]	6.02±0.80	-37.21±3.93[b]	-5.62±0.43
E1562R	-11.38±0.56	7.85±1.22	-53.31±2.39[b]	-7.36±1.05
E1562Q	-3.36±1.60[c]	7.67±0.88	-45.84±2.93	-6.21±0.92

a, $p<0.05$

b, $p<0.01$

c, $p<0.001$

Supplementary Table S2: Primers used to construct the $Na_v1.3$ derived chimeric channels in supplementary Fig S4.

Primer ID	Sequence 5'-3'
1.3/1.5DI-VSD VL-for	CTTAATGGCAATTTTCCTAACAGGGT
1.3/1.5DI-VSD VL-rev	AAGACCATCGTGGGGGCCCTGATCC
1.5DI-VSD S-for	AAAATTGCCATTAAGATTCTGGTTCACTCGCTCTTCAACAT
1.5DI-VSD S-rev	CCCCACGATGGTCTTCAGCCCTGAAATGACTGATATAGTT
1.3/1.5 DI-PD VL-rev	GTCGGACAGCTTCTTCACGGACTGG
1.3/1.5 DI-PD VL-for	TATGAGGAGCAGAACCAGGCCACAC
1.5 DI-PD S-for	AAGAAGCTGTCCGACGTGATGGTCCTCACAGTCTTCTGCC
1.5 DI-PD S-rev	GTTCTGCTCCTCATAGGCCATTGCGACCACGGCCAGGATC
1.3/1.5 DII-VSD VL-rev	ATTCACAAGATGCTTCACTTTTAACCA
1.3/1.5 DII-VSD VL-for	ATGCTCATTAAGATCATCGGCAACTCG
1.5 DII-VSD S-for	AAGCATCTTGTGAATTTGGTGGTCATGGACCCGTTTACTG
1.5 DII-VSD S-rev	GATCTTAATGAGCATCAGGGTGGCCATGATTTGGCCAG
1.3/1.5 DII-PD VL-rev	GTTGCCCAGTGCGCCCACCGAGT
1.3/1.5 DII-PD VL-for	AGTTCCTTTAGTTCAGATAACCTTGC
1.5 DII-PD S-for	GGCGCACTGGGCAACCTGACACTGGTGCTAGCCATCATCG
1.5 DII-PD S-rev	TGAACTAAAGGAACTGAGCAGCAAGGCCAGGAAGAGATTCA
1.3/1.5 DIII-VSDVL-rev	GCTGTAGCATGTCTTCCTAAGATTC
1.3/1.5 DIII-VSD VL-for	GAAGGCATGAGGGTGGTTGTAAATGCTC
1.5 DIII-VSD S-for	AGACATGCTACAGCATCGTGGAGCACAGCTGGTTCGAGA
1.5 DIII-VSD S-rev	CACCCTCATGCCTTCAAATCGTGACAGAGCTCTCAGAG
1.3/1.5 DIII-PD VL-rev	ATTCATGATGGAGGGAATTGCACCAA
1.3/1.5 DIII-PD VL-for	GACAACTTCAACCAGCAGAAGAAGA
1.5 DIII-PD S-for	CCCTCCATCATGAATGTCCTCCTCGTCTGCCTCATCTTCT
1.5 DIII-PD S-rev	CTGGTTGAAGTTGTCAATGATGACACCAATAAAGAGGTTCAG
1.3/1.5 DIV-VSD VL-for	TGAGCCGAGCTTCTTCATTGCATTGT
1.3/1.5 DIV-VSDVL-rev	ACTCTGCTCTTTGCTTTGATGATGT
1.5 DIV-VSD S-for	AAGAAGCTCGGCTCAAAGAAGCCCCAGAAGCCCAT
1.5 DIV-VSD S-rev	AGCAAAGAGCAGAGTGCGGATCCCCTTGGCCCCT
1.3/1.5 DII VL-for	ATTCACAAGATGCTTCACTTTTAACCA
1.3/1.5 DII VL-rev	AGTTCCTTTAGTTCAGATAACCTTGC
1.5 DII S-for	AAGCATCTTGTGAATTTGGTGGTCATGGACCCGTTTACTG
1.5 DII S-rev	TGAACTAAAGGAACTGAGCAGCAAGGCCAGGAAGAGATTCA
1.3/1.5 DIV-PD VL-rev	GTTGAACAGCGCAGGAAGGGACA
1.3/1.5 DIV-PD VL-for	GAGAACTTCAGCGTCGCCACCGAA
1.5 DIV-PD S-for	CCTGCGCTGTTCAAC ATCGGGCTGCTGCTCTTCCTCG
1.5 DIV-PD S-rev	GACGCTGAAGTTCTCCAGGATGATGGCAATGTACATGT

Note: both primers used to linearize the $Na_v1.3$-cloned plasmid and primers used to amplify the specific regions of $Na_v1.5$ are shown. The red labeled regions show the joint sequence.

Supplementary Table S3: Primers used to construct the $Na_v1.5$ derived chimeric channels in supplementary Fig S6

Primer ID	Sequence 5'-3'
1.5/1.3 DI VL-rev	CTTCACAGCCGCTCTCCGGATGGGGT
1.5/1.3 DI VL-for	TATGAGGAGCAAAACCAAGCCACCATC
1.3 DI S-for	AGAGCGGCTGTGAAGATTTTGGTACACTCTTTGTTCAGCA
1.3 DI S-rev	GTTTTGCTCCTCATAGGCCATGGCCACCACA
1.5/1.3 DII VL-rev	CTTCACTCCCTGCTTGATGGACATCCAC
1.5/1.3 DII VL-for	AGCTCCTTCAGTGCAGACAACCTCA
1.3 DII S-for	AAGCAGGGAGTGAAGTTAATTGTGATGGATCCATTTGTTGA
1.3 DII S-rev	TGCACTGAAGGAGCTCAACAATAAGGCCAGAAAGAGGTTCA
1.5/1.3 DIII VL-rev	GTGGTAGCAGGTCTTGCGCAACCGCC
1.5/1.3 DIII VL-for	GACAACTTCAACCAACAGAAGAAAAAG
1.3 DIII S-for	AAGACCTGCTACCACATTGTGGAGCACAACTGGTTTGAGA
1.3 DIII S-rev	TTGGTTGAAGTTGTCTATGATGACACCGATGAATAGATTT
1.5/1.3 DIV VL-rev	GGAGCCCAGCTTCTTCATGGCATT
1.5/1.3 DIV VL-for	GAGAACTTCAGCGTGGCCACGGAGG
1.3 DIV S-for	AAGAAGCTGGGCTCCAAGAAACCTCAGAAGCCCAT
1.3 DIV S-rev	CACGCTGAAGTTCTCCAGGATGACAGCGATGTACATGTTC
1.5/1.3 DII-PD VL-rev	GTTCCCCAGTGCCCCCACTGAGTTC
1.5/1.3 DII-PD VL-for	AGCTCCTTCAGTGCAGACAACCTCA
1.3 DII-PD S-for	GGGGCACTGGGGAACCTGACCCTGGTGCTGGCCATCATCGT
1.3 DII-PD S-rev	TGCACTGAAGGAGCTCAACAATAAGGCCAGAAAGAGGTTCA

Note: both primers used to linearize the $Na_v1.5$-cloned plasmid and primers used to amplify the specific regions of $Na_v1.3$ are shown. The red labeled regions show the joint sequence.

Supplementary Table S4: Protein sequence location of each voltage sensor (VSD)/pore domain (PD) of all four domains of $Na_v1.3$ or $Na_v1.5$.

$Na_v1.3$ domains	Location in $Na_v1.3$	$Na_v1.5$ domains	Location in $Na_v1.5$
$Na_v1.3$ DI-VSD	AA124-AA233	$Na_v1.5$ DI-VSD	AA127-AA236
$Na_v1.3$ DI-PD	AA249-AA426	$Na_v1.5$ DI-PD	AA253-AA415
$Na_v1.3$ DII-VSD	AA706-AA820	$Na_v1.5$ DII-VSD	AA712-AA825
$Na_v1.3$ DII-PD	AA836-AA935	$Na_v1.5$ DII-PD	AA842-AA939
$Na_v1.3$ DIII-VSD	AA1153-AA1269	$Na_v1.5$ DIII-VSD	AA1201-AA1317
$Na_v1.3$ DIII-PD	AA1289-AA1419	$Na_v1.5$ DIII-PD	AA1337-AA1470
$Na_v1.3$ DIV-VSD	AA1473-AA1593	$Na_v1.5$ DIV-VSD	AA1524-AA1644
$Na_v1.3$ DIV-PD	AA1609-AA1722	$Na_v1.5$ DIV-PD	AA1660-AA1772

Supplementary material and methods:

The competitive binding assay:

In Figure 5c and 5d to test the potency of HNTX-III on RTX-VII pretreated $Na_v1.3$ channels, HNTX-III was dissolved in bath solution containing 0.5 uM RTX-VII. Cells transfected with $Na_v1.3$ were incubated with 0.5 μM RTX-VII (total volume: 300 μl) in the recording chamber before treating with HNTX-III of various dose (eg.100 nM HNTX-III). 30 μl of the bath solution in the recording chamber was pipetted out, 30 μl of 1 μM HNTX-III (10 folds concentrated, dissolved in bath solution containing 0.5 μM RTX-VII) was added into the recording chamber far from the recording pipet (the cell) and quickly mixed by repeatedly pipetting to get the final concentration of 100 nM HNTX-III (with a microsyringe connected to the chamber by a thin pipe). To obtain 500 nM final concentration of HNTX-III in the recording chamber on the basis of 100 nM HNTX-III, 30 μl of the bath solution (total volume of 300 μl, already containing 100 nM HNTX-III and 0.5 μM RTX-VII) was pipetted out, and 30 μl of the 4 μM HNTX-III (also dissolved in bath solution containing 0.5 μM RTX-II) was diluted into the chamber far from the recording pipet and quickly mixed by repeatedly pipetting to reach the final concentration of 500 nM HNTX-III. Hence, during the whole time course testing the potency of HNTX-III on $Na_v1.3$, cells were always bathed in 0.5 μM RTX-VII. Note the cover glasses where transfected cells were seeded on were cut into small pieces and were one-use only (if toxin was added, only one cell in one piece of glass was recorded).
